# Supplementary material for: Contribution of primary care expansion to Sustainable Development Goal 3 for health: a microsimulation of the 15 largest cities in Brazil
Source: BMJ Open. 2022 Jan 11;12(1):e049251. doi: 10.1136/bmjopen-2021-049251 (PMC8753407; doi:10.1136/bmjopen-2021-049251)
Supplement: Supplementary data [file bmjopen-2021-049251supp001.pdf]

BMJ Publishing Group Limited (BMJ) disclaims all liability and responsibility arising from any reliance placed on this supplemental material which has been supplied by the author(s)

Supporting Information

Basu S, *et al. BMJ Open* 2022; 12:e049251. doi: 10.1136/bmjopen-2021-049251

Supporting Information Table 1: Hazard ratios for death by cause group, subdivided by sex, race/ethnicity, and whether or not the family receives Bolsa Familia (BF) benefits.

Cause	Subgroup	Hazard Ratio	Lower 95% Confidence interval	Upper 95% Confidence interval
All Causes	Males	0.68	0.64	0.71
All Causes	Females	0.49	0.47	0.52
All Causes	Race White	0.60	0.56	0.64
All Causes	Race Black	0.50	0.47	0.54
All Causes	Race Pardo	0.57	0.54	0.60
All Causes	No Bf	0.64	0.61	0.67
All Causes	Bf	0.52	0.49	0.54
Infectious And Parasitic Diseases	Males	0.82	0.62	1.08
Infectious And Parasitic Diseases	Females	0.53	0.41	0.67
Infectious And Parasitic Diseases	Race White	0.86	0.63	1.17
Infectious And Parasitic Diseases	Race Black	0.68	0.45	1.02
Infectious And Parasitic Diseases	Race Pardo	0.52	0.39	0.69
Infectious And Parasitic Diseases	No Bf	0.68	0.52	0.88
Infectious And Parasitic Diseases	Bf	0.63	0.49	0.82
Hiv/Aids	Males	1.15	0.88	1.52
Hiv/Aids	Females	0.68	0.55	0.83
Hiv/Aids	Race White	0.67	0.44	1.01
Hiv/Aids	Race Black	0.69	0.51	0.93
Hiv/Aids	Race Pardo	1.02	0.80	1.31
Hiv/Aids	No Bf	1.20	0.86	1.68
Hiv/Aids	Bf	0.73	0.61	0.89

Tuberculosis, Malaria, And Ntds	Males	0.91	0.61	1.35
Tuberculosis, Malaria, And Ntds	Females	0.24	0.13	0.43
Tuberculosis, Malaria, And Ntds	Race White	0.61	0.29	1.28
Tuberculosis, Malaria, And Ntds	Race Black	0.49	0.25	0.95
Tuberculosis, Malaria, And Ntds	Race Pardo	0.67	0.42	1.06
Tuberculosis, Malaria, And Ntds	No Bf	0.68	0.38	1.22
Tuberculosis, Malaria, And Ntds	Bf	0.56	0.38	0.82
Maternal Causes (Females Only)	Females	0.97	0.60	1.56
Maternal Causes (Females Only)	Race White	1.01	0.46	2.23
Maternal Causes (Females Only)	Race Black	1.09	0.46	2.56
Maternal Causes (Females Only)	Race Pardo	0.82	0.37	1.82
Maternal Causes (Females Only)	No Bf	1.93	0.63	5.90
Maternal Causes (Females Only)	Bf	0.81	0.48	1.39
Nutritional Deficiencies	Males	0.59	0.26	1.33
Nutritional Deficiencies	Females	0.52	0.28	0.98
Nutritional Deficiencies	Race White	0.21	0.05	0.97
Nutritional Deficiencies	Race Black	0.40	0.14	1.19
Nutritional Deficiencies	Race Pardo	0.92	0.50	1.69
Nutritional Deficiencies	No Bf	0.99	0.53	1.88
Nutritional Deficiencies	Bf	0.23	0.10	0.54
Diseases Of The Nervous System	Males	0.52	0.28	0.98
Diseases Of The Nervous System	Females	0.58	0.38	0.91
Diseases Of The Nervous System	Race White	0.46	0.25	0.88

Diseases Of The Nervous System	Race Black	0.83	0.40	1.75
Diseases Of The Nervous System	Race Pardo	0.55	0.31	0.97
Diseases Of The Nervous System	No Bf	0.48	0.29	0.82
Diseases Of The Nervous System	Bf	0.68	0.41	1.12
Endocrine Disorders	Males	0.74	0.57	0.95
Endocrine Disorders	Females	0.50	0.41	0.61
Endocrine Disorders	Race White	0.58	0.43	0.79
Endocrine Disorders	Race Black	0.55	0.40	0.74
Endocrine Disorders	Race Pardo	0.60	0.47	0.76
Endocrine Disorders	No Bf	0.59	0.47	0.75
Endocrine Disorders	Bf	0.59	0.47	0.73
Mental And Substance Use Disorders	Males	0.66	0.41	1.05
Mental And Substance Use Disorders	Females	0.27	0.15	0.50
Mental And Substance Use Disorders	Race White	0.67	0.32	1.41
Mental And Substance Use Disorders	Race Black	0.17	0.05	0.56
Mental And Substance Use Disorders	Race Pardo	0.53	0.32	0.86
Mental And Substance Use Disorders	No Bf	0.75	0.36	1.54
Mental And Substance Use Disorders	Bf	0.40	0.26	0.61
Stroke	Males	0.77	0.63	0.94
Stroke	Females	0.45	0.38	0.53
Stroke	Race White	0.67	0.53	0.85
Stroke	Race Black	0.45	0.34	0.58
Stroke	Race Pardo	0.60	0.50	0.73

Basu S, *et al. BMJ Open* 2022; 12:e049251. doi: 10.1136/bmjopen-2021-049251

Stroke	No Bf	0.65	0.54	0.78
Stroke	Bf	0.50	0.42	0.60
Heart Disease	Males	0.59	0.52	0.67
Heart Disease	Females	0.41	0.37	0.46
Heart Disease	Race White	0.47	0.40	0.56
Heart Disease	Race Black	0.43	0.35	0.52
Heart Disease	Race Pardo	0.51	0.44	0.58
Heart Disease	No Bf	0.52	0.46	0.59
Heart Disease	Bf	0.47	0.42	0.54
Other Cardiovascular Diseases	Males	0.68	0.54	0.86
Other Cardiovascular Diseases	Females	0.49	0.41	0.60
Other Cardiovascular Diseases	Race White	0.63	0.47	0.84
Other Cardiovascular Diseases	Race Black	0.41	0.30	0.56
Other Cardiovascular Diseases	Race Pardo	0.65	0.52	0.81
Other Cardiovascular Diseases	No Bf	0.64	0.52	0.79
Other Cardiovascular Diseases	Bf	0.50	0.40	0.62
Digestive Diseases	Males	0.67	0.53	0.84
Digestive Diseases	Females	0.52	0.41	0.66
Digestive Diseases	Race White	0.61	0.46	0.83
Digestive Diseases	Race Black	0.47	0.32	0.70
Digestive Diseases	Race Pardo	0.61	0.48	0.79
Digestive Diseases	No Bf	0.61	0.47	0.79
Digestive Diseases	Bf	0.59	0.48	0.74
Genitourinary Diseases	Males	0.67	0.51	0.88

Genitourinary Diseases	Females	0.52	0.42	0.65
Genitourinary Diseases	Race White	0.63	0.46	0.87
Genitourinary Diseases	Race Black	0.60	0.43	0.83
Genitourinary Diseases	Race Pardo	0.57	0.44	0.74
Genitourinary Diseases	No Bf	0.64	0.51	0.80
Genitourinary Diseases	Bf	0.52	0.40	0.68
Unintentional Injuries	Males	0.56	0.43	0.74
Unintentional Injuries	Females	0.43	0.32	0.58
Unintentional Injuries	Race White	0.50	0.33	0.76
Unintentional Injuries	Race Black	0.47	0.31	0.71
Unintentional Injuries	Race Pardo	0.53	0.40	0.71
Unintentional Injuries	No Bf	0.55	0.40	0.78
Unintentional Injuries	Bf	0.46	0.36	0.60
Intentional Injuries	Males	0.42	0.33	0.53
Intentional Injuries	Females	0.30	0.18	0.50
Intentional Injuries	Race White	0.51	0.33	0.79
Intentional Injuries	Race Black	0.45	0.27	0.73
Intentional Injuries	Race Pardo	0.34	0.24	0.46
Intentional Injuries	No Bf	0.72	0.51	1.01
Intentional Injuries	Bf	0.28	0.21	0.37
Neoplasms	Males	0.97	0.86	1.11
Neoplasms	Females	0.61	0.55	0.68
Neoplasms	Race White	0.68	0.59	0.78
Neoplasms	Race Black	0.69	0.57	0.84
Neoplasms	Race Pardo	0.83	0.74	0.93

Neoplasms	No Bf	0.77	0.69	0.86
Neoplasms	Bf	0.73	0.65	0.82
Respiratory Infections And Diseases	Males	0.84	0.73	0.98
Respiratory Infections And Diseases	Females	0.49	0.42	0.56
Respiratory Infections And Diseases	Race White	0.73	0.62	0.87
Respiratory Infections And Diseases	Race Black	0.57	0.44	0.72
Respiratory Infections And Diseases	Race Pardo	0.58	0.49	0.69
Respiratory Infections And Diseases	No Bf	0.68	0.59	0.79
Respiratory Infections And Diseases	Bf	0.61	0.52	0.70

Supporting Information Table 2: (A) 95% lower confidence interval estimates and (B) 95% upper confidence interval estimates around the relative impact on cause-specific mortality given changes in the percentage point coverage in the FHS program (Estratégia de Saúde da Família) primary care program. The cells show the ratio of mortality by cause under different levels of FHS coverage, compared to the current mortality rate (at 0% change in FHS coverage), the reference column.

(A)

	Ratio of mortality by cause, compared to current mortality rate (at 0%)						
**Percentage point change in FHS coverage:**	**-20%**	**-10%**	**0%**	**10%**	**20%**	**30%**	**40%**
All causes	1.07	1.00	0.93	0.86	0.79	0.72	0.65
Infections (excluding HIV, TB, malaria, NTDs)	1.07	1.00	0.93	0.86	0.79	0.72	0.66
HIV	1.04	0.99	0.93	0.88	0.82	0.76	0.70
TB, malaria, NTDs	1.00	0.97	0.93	0.90	0.86	0.82	0.78
Respiratory	1.06	1.00	0.93	0.87	0.80	0.73	0.67
Nutrition	1.05	0.99	0.93	0.87	0.81	0.75	0.69
Neoplasms	1.07	1.00	0.93	0.86	0.79	0.72	0.65
Nervous system	1.02	0.98	0.93	0.89	0.84	0.79	0.74
Endocrine	1.07	1.00	0.93	0.86	0.79	0.72	0.65
Mental/substance use	1.07	1.00	0.93	0.86	0.80	0.73	0.66
Stroke	1.10	1.01	0.93	0.85	0.77	0.69	0.61
Heart disease	1.07	1.00	0.93	0.86	0.80	0.73	0.66
Other cardiovascular	1.09	1.01	0.93	0.85	0.77	0.70	0.62
Digestive	1.07	1.00	0.93	0.86	0.80	0.73	0.66
Genitourinary	1.06	1.00	0.93	0.87	0.80	0.74	0.67
Unintentional injuries	1.09	1.01	0.93	0.85	0.77	0.69	0.62
Intentional injuries	1.13	1.03	0.93	0.84	0.74	0.65	0.56
Maternal	0.97	0.95	0.93	0.91	0.88	0.85	0.82

(B)

	**Ratio of mortality by cause, compared to current mortality rate (at 0%)**						
**Percentage point change in FHS coverage:**	**-20%**	**-10%**	**0%**	**10%**	**20%**	**30%**	**40%**
All causes	1.15	1.11	1.07	1.03	0.99	0.95	0.91
Infections (excluding HIV, TB, malaria, NTDs)	1.14	1.11	1.07	1.03	0.99	0.96	0.92
HIV	1.11	1.09	1.07	1.05	1.02	1.00	0.98
TB, malaria, NTDs	1.07	1.07	1.07	1.07	1.08	1.08	1.09
Respiratory	1.14	1.10	1.07	1.03	1.00	0.97	0.94
Nutrition	1.12	1.09	1.07	1.04	1.02	0.99	0.97
Neoplasms	1.14	1.11	1.07	1.03	0.99	0.95	0.91
Nervous system	1.09	1.08	1.07	1.06	1.05	1.04	1.04
Endocrine	1.14	1.11	1.07	1.03	0.99	0.96	0.92
Mental/substance use	1.14	1.10	1.07	1.03	1.00	0.96	0.93
Stroke	1.17	1.12	1.07	1.02	0.96	0.91	0.85
Heart disease	1.14	1.11	1.07	1.03	0.99	0.96	0.92
Other cardiovascular	1.17	1.12	1.07	1.02	0.97	0.92	0.86
Digestive	1.14	1.10	1.07	1.03	1.00	0.96	0.92
Genitourinary	1.13	1.10	1.07	1.04	1.00	0.97	0.94
Unintentional injuries	1.17	1.12	1.07	1.02	0.97	0.92	0.86
Intentional injuries	1.20	1.14	1.07	1.00	0.93	0.86	0.78
Maternal	1.04	1.05	1.07	1.09	1.10	1.13	1.15

Basu S, *et al. BMJ Open* 2022; 12:e049251. doi: 10.1136/bmjopen-2021-049251

Supporting Information Figure 1: Hazard ratios for FHS users (compared to non-users) by cause of death.

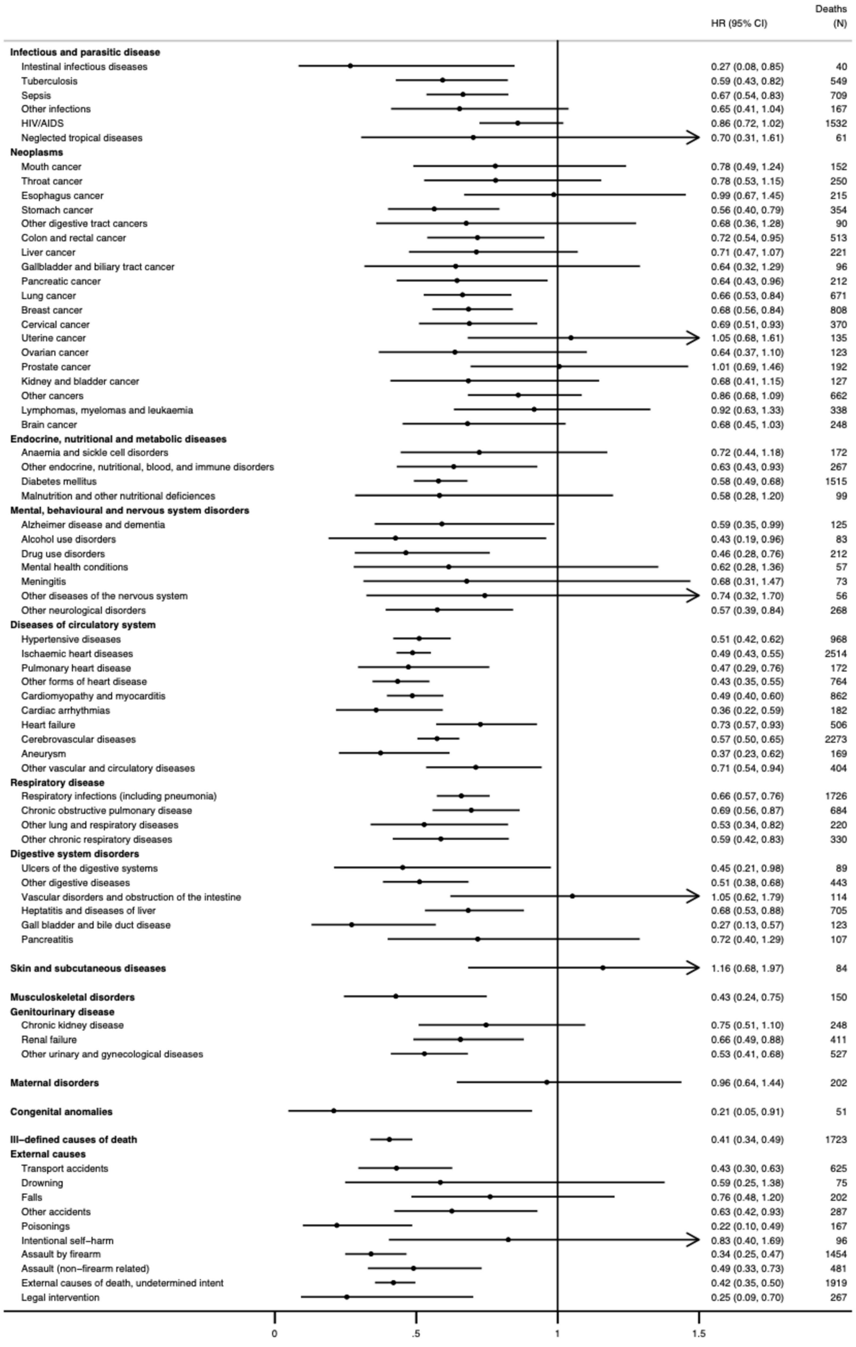

Supporting Information Figure 2: Uncertainty estimates around mean projected variations in (A) all-cause crude mortality, (B) all-cause age-standardized mortality, (C) infant mortality and (D) under-5 mortality given different levels of Family Health Strategy (FHS) program primary care coverage. See Table 1 for current coverage levels corresponding to a 0% change on the x-axis. Mean values are population-weighted across all 15 Brazilian cities.

(A)

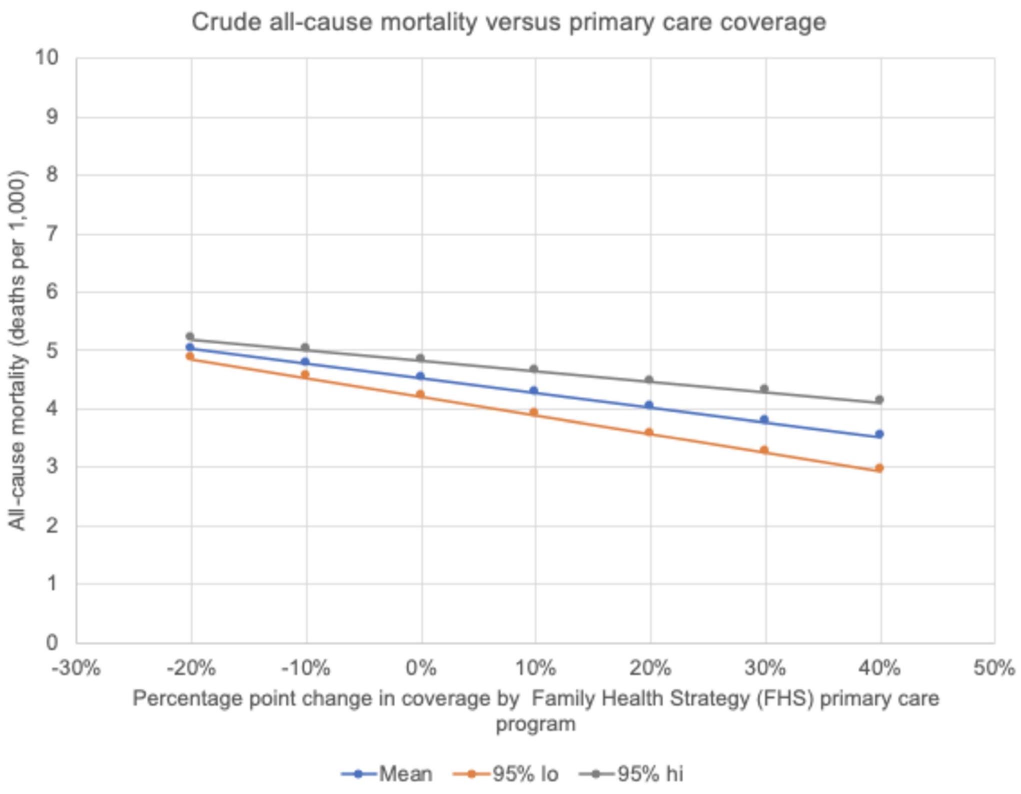

(B)

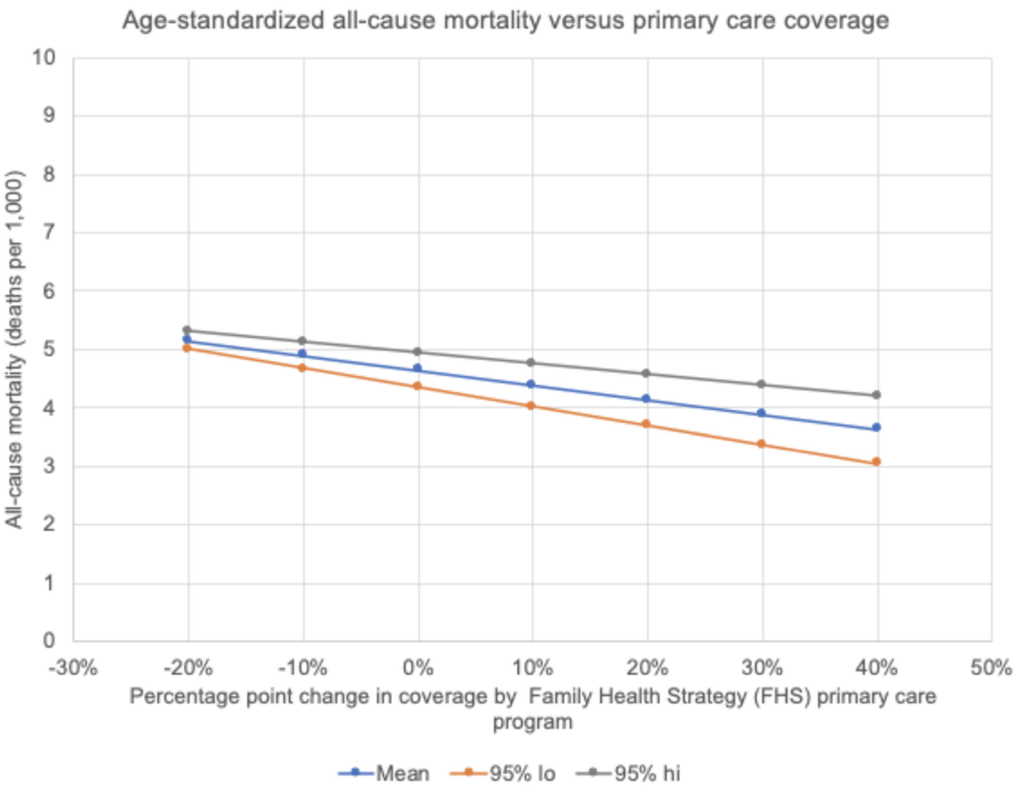

Basu S, *et al. BMJ Open* 2022; 12:e049251. doi: 10.1136/bmjopen-2021-049251

(C)

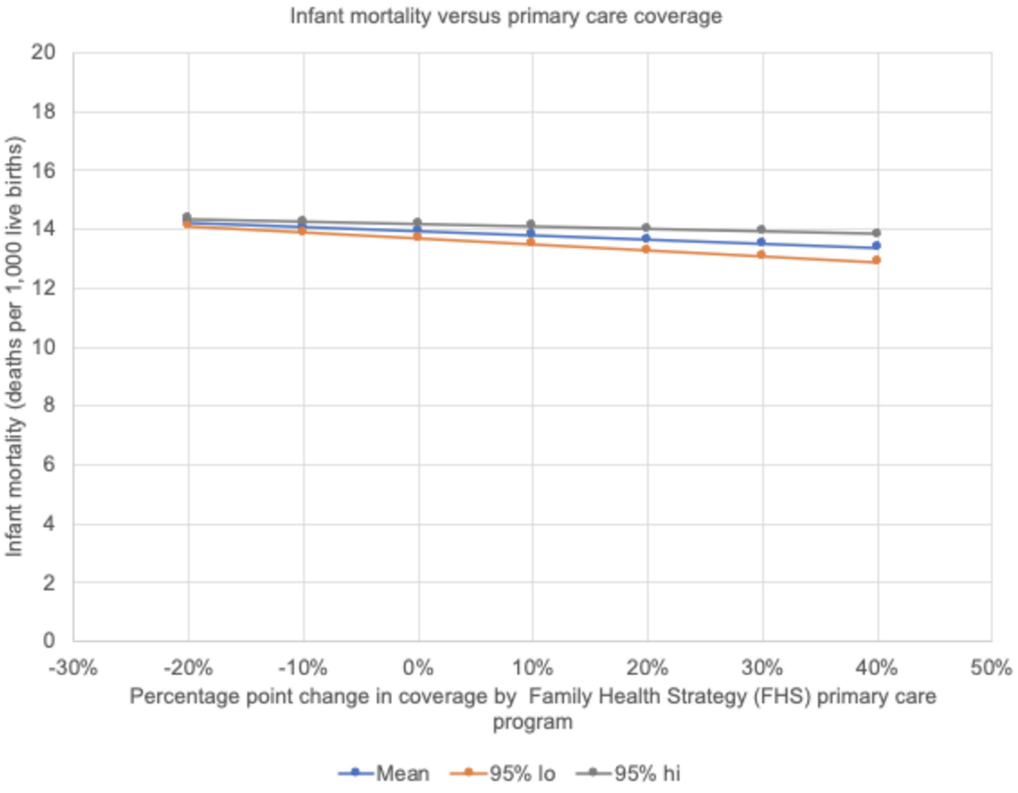

Basu S, *et al. BMJ Open* 2022; 12:e049251. doi: 10.1136/bmjopen-2021-049251

(D)

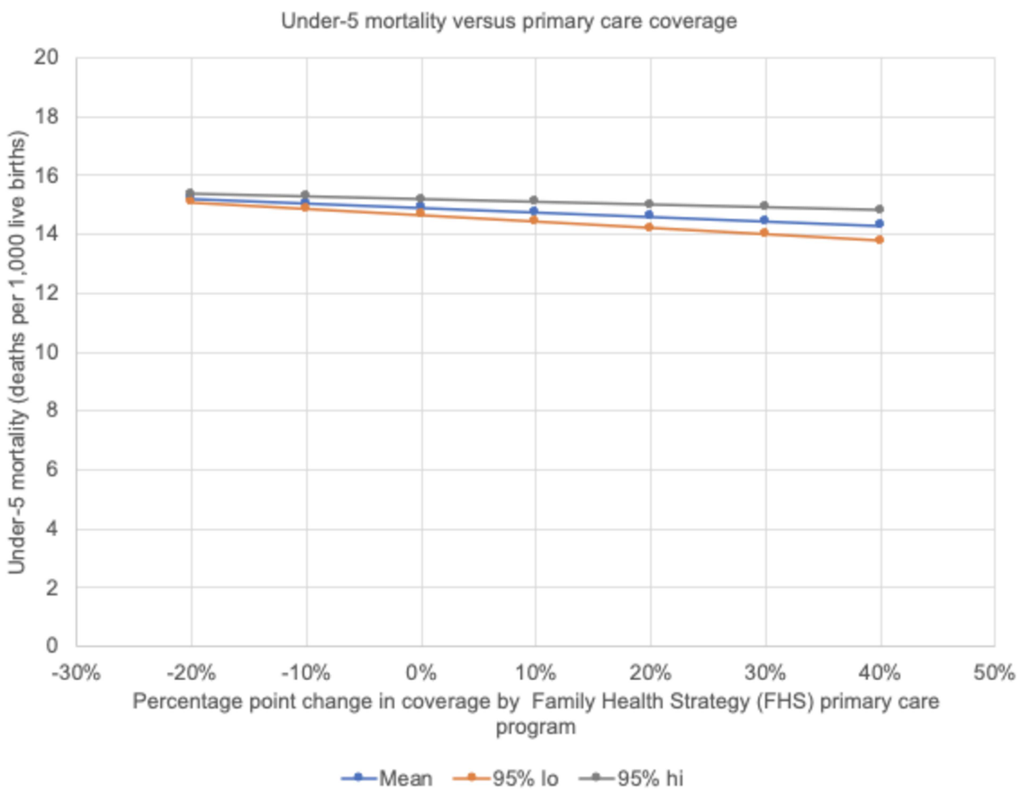
Under-5 mortality versus primary care coverage
Under-5 mortality (deaths per 1,000 live births)
20
18
16
14
12
10
8
6
4
2
0
-30%
-20%
-10%
0%
10%
20%
30%
40%
50%
Percentage point change in coverage by Family Health Strategy (FHS) primary care program
Mean
95% lo
95% hi
