# Supplementary material for: Contribution of primary care expansion to Sustainable Development Goal 3 for health: a microsimulation of the 15 largest cities in Brazil
Source: BMJ Open. 2022 Jan 11;12(1):e049251. doi: 10.1136/bmjopen-2021-049251 (PMC8753407; doi:10.1136/bmjopen-2021-049251)
Supplement: Author's manuscript [file bmjopen-2021-049251.draft_revisions.pdf]

BMJ Open

BMJ Open is committed to open peer review. As part of this commitment we make the peer review history of every article we publish publicly available.

When an article is published we post the peer reviewers' comments and the authors' responses online. We also post the versions of the paper that were used during peer review. These are the versions that the peer review comments apply to.

The versions of the paper that follow are the versions that were submitted during the peer review process. They are not the versions of record or the final published versions. They should not be cited or distributed as the published version of this manuscript.

BMJ Open is an open access journal and the full, final, typeset and author-corrected version of record of the manuscript is available on our site with no access controls, subscription charges or pay-per-view fees (http://bmjopen.bmj.com).

If you have any questions on BMJ Open's open peer review process please email info.bmjopen@bmj.com

BMJ Open

The contribution of primary care expansion to Sustainable Development Goal Three for health: A microsimulation of the fifteen largest cities in Brazil

Journal:	*BMJ Open*
Manuscript ID	bmjopen-2021-049251
Article Type:	Original research
Date Submitted by the Author:	23-Jan-2021

Complete List of Authors:	Basu, Sanjay; Harvard Medical School,<br>Hone, Thomas; Imperial College London<br>Villela, Daniel; Oswaldo Cruz Foundation<br>Saraceni, Valeria; FIOCRUZ<br>Trajman, Anete; Universidade Gama Filho<br>Durovni, Betina; Fundação Oswaldo Cruz<br>Millett, Christopher; Imperial College London, Primary Care and Social Medicine<br>Rasella, Davide; Federal University of Bahia

Keywords:	PRIMARY CARE, PUBLIC HEALTH, GENERAL MEDICINE (see Internal Medicine)

SCHOLARONE™
Manuscripts

I, the Submitting Author has the right to grant and does grant on behalf of all authors of the Work (as defined in the below author licence), an exclusive licence and/or a non-exclusive licence for contributions from authors who are: i) UK Crown employees; ii) where BMJ has agreed a CC-BY licence shall apply, and/or iii) in accordance with the terms applicable for US Federal Government officers or employees acting as part of their official duties; on a worldwide, perpetual, irrevocable, royalty-free basis to BMJ Publishing Group Ltd ("BMJ") its licensees and where the relevant Journal is co-owned by BMJ to the co-owners of the Journal, to publish the Work in this journal and any other BMJ products and to exploit all rights, as set out in our licence.

The Submitting Author accepts and understands that any supply made under these terms is made by BMJ to the Submitting Author unless you are acting as an employee on behalf of your employer or a postgraduate student of an affiliated institution which is paying any applicable article publishing charge ("APC") for Open Access articles. Where the Submitting Author wishes to make the Work available on an Open Access basis (and intends to pay the relevant APC), the terms of reuse of such Open Access shall be governed by a Creative Commons licence – details of these licences and which Creative Commons licence will apply to this Work are set out in our licence referred to above.

Other than as permitted in any relevant BMJ Author's Self Archiving Policies, I confirm this Work has not been accepted for publication elsewhere, is not being considered for publication elsewhere and does not duplicate material already published. I confirm all authors consent to publication of this Work and authorise the granting of this licence.

For peer review only

The contribution of primary care expansion to Sustainable Development Goal Three for health: A microsimulation of the fifteen largest cities in Brazil

Sanjay Basu MD PhD[1,2,3,4*], Thomas V. Hone PhD[1], Daniel Villela[5], Valeria Saraceni[6], Anete Trajman[7], Betina Durovni[8], Christopher Millett PhD[1,9,10], Davide Rasella PhD[11,1]

[1] Public Health Policy Evaluation Unit, Imperial College London, London, UK

[2] Center for Primary Care, Harvard Medical School, Boston, MA

[3]Ariadne Labs, Brigham and Women's Hospital and Harvard T.H. Chan School of Public Health, Boston, MA

[4] Research and Population Health, Collective Health, San Francisco, CA

[5] Program of Scientific Computing, Oswaldo Cruz Foundation, Rio de Janeiro, Brazil

[6] Secretaria Municipal de Saúde do Rio de Janeiro, Rio de Janeiro, Brazil

[7] Centro de Estudos Estratégicos, Oswaldo Cruz Foundation, Rio de Janeiro, Brazil

[8] Programa de Pós-graduação em Clínica Médica and Mestrado Profissional em Atenção Primária à Saúde, Federal University of Rio de Janeiro, Brazil

[9] Department of Preventive Medicine, School of Medicine, University of São Paulo, São Paulo, Brazil

[10] Center of Data and Knowledge Integration for Health (CIDACS), Instituto Gonçalo Muniz, Fundação Oswaldo Cruz, Salvador, Brazil

[11] ISGlobal, Hospital Clínic - Universitat de Barcelona, Barcelona, Spain

* to whom correspondence should be addressed:

Sanjayb493@gmail.com

635 Huntington Avenue

Second Floor

Boston, MA 02115

(617) 432-2222

Word count: 3,445/4,000

For peer review only

For peer review only - http://bmjopen.bmj.com/site/about/guidelines.xhtml

Abstract

Objectives

As middle-income countries strive to achieve the Sustainable Development Goals (SDGs), it remains unclear to what degree expanding primary care coverage can help achieve those goals and reduce within-country inequalities in mortality. Our objective was to estimate the potential impact of primary care expansion on cause-specific mortality in the 15 largest Brazilian cities.

Design

Microsimulation model

Setting

15 largest cities by population size in Brazil

Participants

Simulated populations

Interventions

We estimated hazard ratios of death by cause and by demographic group, from a national administrative database linked to the Estratégia de Saúde da Família (Family Health Strategy, FHS) electronic health and death records among 1.2 million residents of Rio de Janeiro (2010-2016). We incorporated these hazard ratios into a microsimulation to estimate the impact of changing primary care coverage in the 15 largest cities by population size in Brazil.

Primary and secondary outcome measures

Crude and age-standardized mortality by cause, infant mortality, and under-5 mortality.

Results

Increased FHS coverage would be expected to reduce inequalities in mortality among cities (from 2.8 to 2.4 deaths per 1,000 between the highest- and lowest-mortality city, given a 40 percentage point increase in coverage), between welfare recipients and non-recipients (from 1.3 to 1.0 deaths per 1,000), and among race/ethnic groups (between Black and White Brazilians from 1.0 to 0.8 deaths per 1,000). Even a 40 percentage point increase in coverage, however, would be insufficient to reach SDG targets alone--reducing premature mortality from non-communicable diseases by 20% (versus the target of 33%), and communicable diseases by 15% (versus 100%).

For peer review only - http://bmjopen.bmj.com/site/about/guidelines.xhtml

Conclusions

FHS primary care coverage may be critically beneficial to reducing within-country health inequalities, but reaching SDG targets will likely require coordination between primary care and other sectors.

For peer review only

For peer review only - http://bmjopen.bmj.com/site/about/guidelines.xhtml

Strengths and limitations of this study

This study quantified the degree to which expansion or contraction of Brazil's largest primary care program would be expected to help achieve the SDGs, and the implications of program expansion or contraction on inequalities.

The findings suggest that primary care coverage may be critically beneficial to reducing within-country health inequalities, but reaching he Sustainable Development Goal (SDG) targets would be unlikely without additional resources and efforts from other sectors.

The study helps direct emphasis towards coordination between primary care and other sectors, and efforts to address the wider socioeconomic determinants of health.

The principal limitations arise from being based on a simulation model that cannot account for unobserved confounders.

Additionally, the infant mortality and under-5 mortality outcomes were based on hazard ratios from the literature rather than from detailed individual-level data, due to limitations in data availability from the primary datasets we used.

For peer review only - http://bmjopen.bmj.com/site/about/guidelines.xhtml

Introduction

Increasing access to primary care has been linked to reduced mortality at both the individual and population levels.[1–7] Primary care expansion in low- and middle-income countries remains a major strategy for reducing mortality, and for achieving the United Nations Sustainable Development Goals (SDGs).[8] Indeed, primary care expansion is listed by the United Nations as a key intervention to achieve the SDG mortality reduction targets, including reducing premature mortality from non-communicable diseases by one-third, ending deaths from communicable diseases, and reducing under-5 mortality to <25 per 1,000 live births by the year 2030.[9]

Yet the degree to which improving primary care access can further contribute to reductions in mortality remains unclear. Additionally, better evidence is needed to drive policy-making given local health system planning and budgeting is currently out of sync with global mortality targets and are often insufficiently tailored to local contexts.[10,11] Primary care divestments by national governments, seen in middle-income countries such as Brazil during recent recessions, have partially been justified by ambiguity over how much primary care investments can be expected to achieve targets such as the SDGs.[12–14] Local governments often vary in their ability and willingness to fund primary care. Hence, particularly in countries with decentralized decision-making, it is vital to estimate expected declines in mortality from primary care investments, and conversely whether targets can be better designed with local and regional baseline conditions and primary care effectiveness in mind.[15]

In this study, we estimated the potential impact of primary care expansion on mortality by cause of death and by age in the 15 largest Brazilian cities.We use a simulation model incorporating micro-level demographic, health, and effect-size data from Brazil's Estratégia de Saúde da Família (Family Health Strategy, FHS) primary care program. The FHS is Brazil's main strategy for achieving universal health care, and is based on primary care delivery through multidisciplinary care teams co-located in clinics; mobile community healthcare worker teams trained to extend clinic reach; and evidence-based training, protocol management, and record-keeping systems (in Rio de Janeiro) including a unique administrative dataset for tracking individual FHS users and non-users and their health outcomes.[1,2] Each team includes a physician,

For peer review only - http://bmjopen.bmj.com/site/about/guidelines.xhtml

nurses and community healthcare workers responsible for delivering maternal and child healthcare, curative care, health promotion and prevention, chronic disease management, home visits, and referrals to a catchment population of approximately 1,000 families (~3,450 individuals). In 2020, 5,462 local government municipalities (out of 5,565 in Brazil) had these health teams, covering 133.9 million individuals (63.7% of the population).[16] Since 1996, municipal governments have been responsible for financing and delivery of primary care in Brazil. In the context of national government cuts to healthcare budgets, local governments have varied in the degree to which they augment primary care investments.[12,13]

Our prior work has shown that expanded FHS coverage in Brazil has been associated with reductions in both non-communicable and communicable disease mortality, infant and under-5 mortality, as well as health disparities among race/ethnic and urban/rural groups.[1,2,7,12] Here, we simulated the fifteen largest Brazilian cities with detailed demographic and health data--incorporating information on variations in FHS primary care coverage, and estimated relationships among coverage to mortality risk at an individual level by cause--to project how further expansion or contraction of the FHS program may affect crude and age-standardized mortality by cause, infant mortality, and under-5 mortality, and to compare such mortality rate variations to international targets for mortality. We focused on cities because they have an average FHS coverage of 35% -- far below the national coverage --yet constitute the largest proportion of under-served favela (slum) populations who are intended primary target populations for FHS.[1]

For peer review only - http://bmjopen.bmj.com/site/about/guidelines.xhtml

Methods

Model structure

We designed and implemented a microsimulation model,[17] which simulates individual people in each of the largest fifteen Brazilian cities, their demographics, their risk of specific causes of death conditional on their location and demographics, and the estimated change in their risk of death given expansion or contraction of the FHS primary care program, over the period 2020-2030. A microsimulation is a model that can be envisioned as a large table, where each row is an individual and each column is a characteristic (i.e., location, demographic, health status) of the individual. Within the microsimulation, probabilities of death by cause by year (derived from annual mortality rate by cause) are conditioned on the location and demographics of the individual, as well as whether or not they have access to the FHS primary care program. We adjusted those probabilities of death to simulate changes in FHS coverage, per the data sources detailed below.

Relationship between FHS primary care coverage and mortality outcomes

FHS effects estimates were obtained from a previous retrospective study on the association between FHS usage and mortality by age-groups, socioeconomic groups, and causes of death for 1.2 million residents of Rio de Janeiro (2010-2016) .[18] his evaluation was carried out using linkages between *Cadastro Único* national administrative database,[19] FHS electronic health records and mortality records. g . Flexible parametric survival analysis models were utilized to estimate hazard ratios by sex, race, and if the family receives benefits from the national conditional cash transfers program Bolsa Familia or not. Inverse probability treatment weighting and regression adjustment (IPTW-RA) was used to maximize causal inference. Results are displayed in **Supporting Information Figure 1**. By contrast with cause-specific adult mortality, hazard ratios for infant mortality and under-5 mortality were obtained from a prior systematic review of literature, as routine administrative data is not disaggregated sufficiently.[21]

Simulation of changing FHS primary care coverage levels

For peer review only - http://bmjopen.bmj.com/site/about/guidelines.xhtml

We used data from the Brazilian Institute of Geography and Statistics (IBGE) on fifteen Brazilian cities (including Rio de Janeiro) to generate a simulated population of each city.[22] We generated the simulated representative population based on the demographic characteristics, FHS enrollment probability and mortality risks of each population, itemized in **Table 1**. We then varied the FHS coverage level in each city, and applied the hazard ratios estimated from the Rio de Janeiro data analysis to estimate the impact of changing FHS coverage on mortality in all of the simulated cities, through the method specified below.

We specifically estimated the impact of changing coverage on mortality in two steps. First, we calculated the base mortality probability for each simulated person in the absence of FHS primary care, using the following formula:

[Eq. 1] $m_b \times HR \times p + m_b \times (1 - p) = m_c$,

where $m_b$is the base mortality probability for each cause of death in each city without FHS (to be estimated); *HR* is the hazard ratio for that cause of death for FHS users versus non-users obtained from the Rio de Janeiro analysis (described above); *p* is the latest (2016 observed) FHS-covered proportion of the population in the simulated city, and $m_c$is the latest (2016 observed) mortality probability in that city. We used 2016 because it was the last year of data for the Rio de Janeiro analysis.[18]

Second, after calculating the base mortality probability for each cause of death in each city, we then estimated the new mortality probability from equation 1, conditional on a simulated FHS coverage level, to re-estimate the deaths by cause under different FHS coverage levels. We varied the FHS coverage level from 20 percentage points below the current observed coverage level to 40 percentage points above the current observed coverage level in each city (up to a maximum of 100 percent). The choice of 40 percentage points was based on the assumption that the FHS expansion policy would have been homogeneously coordinated at the federal level, with an average effort to progress from the 60% FHS national coverage to a full 100% coverage by 2030.

The primary outcome was change in all-cause mortality. Secondary outcomes included changes in cause-specific and all-cause mortality subgrouped by race/ethnicity and whether the family receives Bolsa Familia benefits or not, taking into account the differential hazard ratios of death by cause and by these characteristics, as described above.

For peer review only - http://bmjopen.bmj.com/site/about/guidelines.xhtml

Uncertainty analyses

At each level of simulated FHS coverage, we repeatedly simulated each of the fifteen cities' populations a total of 10,000 times each. During each of these simulations, we sampled with replacement from random normal distributions constructed around the mean and 95% confidence intervals around the mean of the hazard ratios of FHS primary care enrollment on each cause of death (**Supporting Materials Figure 1, Supporting Materials Table 1**), to generate uncertainty intervals around our outcome estimates. All analyses were performed in *R*. Approval for this study was obtained from the Brazilian National Commission for Ethics in Research (Comissão Nacional de Ética em Pesquisa [CONEP]; number 2.689.528) and Imperial College London's Ethical Committee.

Role of the funding sources

This work was supported by a grant from the Health Systems Research Initiative with funding from the Foreign, Commonwealth and Development Office, the UK Medical Research Council and Wellcome, in collaboration with the UK Economic and Social Research Council (grant no. MR/P014593/1). The funder had no role in study design; collection, analysis, and interpretation of data; report writing; or decision to submit the paper for publication.
Patients/public were not involved in the research.

Patient and public involvement

No patient involved.

For peer review only

For peer review only - http://bmjopen.bmj.com/site/about/guidelines.xhtml

Results

Population characteristics

Population demographics, FHS primary care program coverage rates, and mortality rate estimates by city are provided in **Table 1**. Notable demographics included the population aged less than 15 years (which varied between 18.8% to 29.9%), the populations aged above age 64 years (between 3.2% and 7.7%), race/ethnicity (varying, between 14.8% and 78.0% classifying as white, between 2.3% and 38.0% classifying as black, and 10.2% to 77.0% classifying Pardo). City poverty rates varied from 1.7% to 15.6%.Age-standardized all-cause mortality rates varied from 4.86 to 7.04 per 1,000 population, infant mortality varied from 11.8 to 22.0 per 1,000 live births, and under-5 mortality from 12.0 to 24.0 per 1,000 live births. (hence, all cities had rates below 25 per 1,000 live births meeting SDG target 3.2).

Projected mortality variations with changes in primary care coverage

In our simulations, we varied the FHS coverage level from 20 percentage points below the current observed coverage level to 40 percentage points above the current observed coverage level in each city (up to a maximum of 100 percent). Projected variations in crude and age-standardized all-cause mortality, as well as in infant mortality and under-5 mortality, are illustrated in **Figures 1 and 2**. Variations in cause-specific age-standardized mortality are itemized in **Table 2**. Uncertainty estimates (95% confidence intervals) around all estimates are provided in **Supporting Information Figure 2** and **Supporting Information Table 2**. In general, increases in FHS coverage were associated with reductions in predicted mortality.

As shown in **Figure 1**, increases in FHS coverage would contribute to reductions in inequalities between cities in both crude- and all-cause mortality. The associations between FHS coverage and reductions in mortality were greatest in cities with the highest rates of baseline mortality. For example, a 20 percentage point decline in coverage would increase crude mortality in Rio de Janeiro by 12% (from a current level of 5.9 to 6.6 per 1,000 [95% CI: 6.2, 7.0]), whereas a 20 percentage point decline increase crude mortality in Sao Paulo by 10% (from 4.8 to 5.3 per 1,000 [95% CI: 5.2, 5.4]). By contrast, as shown in **Figure 1**, when primary care coverage rates increased, the model observed less inequality among cities, with diminishing

For peer review only - http://bmjopen.bmj.com/site/about/guidelines.xhtml

returns on further reductions in mortality among those already with low mortality rates. For example, a 20 percentage point increase in coverage would be expected to reduce crude mortality by 21% in Sao Paulo (from 4.8 to 3.8 per 1,000 [95% CI: 3.3, 4.4]) versus by 24% in Rio de Janeiro (from 5.9 to 4.5 per 1,000 [95% CI: 3.4, 5.5]). A 40 percentage point increase in coverage would be expected to reduce overall between-city all-cause mortality (differences in mortality between the highest and lowest mortality city) from 2.8 to 2.4 deaths per 1,000 (95% CI: 2.3, 2.6). The same phenomenon was observed in infant mortality (**Figure 1C**) and under-5 mortality (**Figure 1D**).

As shown in **Table 2**, the specific causes of mortality that were most sensitive to the changes in FHS primary care coverage were chronic non-communicable disease deaths, including cardiovascular disease deaths and deaths from injury (likely because many FHS clinics are often the first point of service for injuries). According to the model, a 20 percentage point reduction in coverage would be expected to raise deaths from unintentional and intentional injuries by 13% (95% CI: 9%, 17%) and 16% (95% CI: 13%, 20%), respectively, and from heart disease and stroke from 11% (95% CI: 7%, 14%) and 14% (95% CI: 10%, 17%), respectively. Conversely,the smallest changes in cause-specific mortality would be for deaths from nervous system diseases, tuberculosis, malaria, neglected tropical diseases, and maternal deaths. For the SDG of reducing by one-third premature mortality (death prior to age 70 years) from non-communicable diseases, the results imply that even increases in FHS primary care coverage by 40 percentage points would still insufficient by itself to reach the target, as premature mortality from non-communicable diseases is only estimated to fall by 20% (95% CI: 7%, 34%). For the SDG of ending deaths from communicable diseases, the model result implies that even an increase in FHS primary care coverage by 40 percentage points would be expected to reduce mortality from communicable diseases by 15% (95% CI: 1%, 29%).

Subgroup analyses

The model was used to estimate changes in mortality across groups defined by race/ethnicity and whether or not the family receives Bolsa Familia benefits, taking into account the differential hazard ratios of death by cause and by these characteristics (**Supporting Information Table 1**). Across all causes of death, FHS primary care coverage disproportionately

For peer review only - http://bmjopen.bmj.com/site/about/guidelines.xhtml

benefited Black and Pardo groups, and those on Bolsa Familia benefits. For each percentage point decline (or increase) in primary care FHS coverage, the absolute increase (or decrease) in mortality was 1.3 times higher for Bolsa Familia families than for non-recipient families, 1.2 times higher for Black and 1.1 times higher for Pardo families than for White families (**Figure 2**). A 40 percentage point increase in coverage would be expected to reduce inequality in mortality between welfare recipients and non-recipients from 1.3 to 1.0 deaths per 1,000 (95% CI: 0.8, 1.2), inequality between Black and White Brazilians from 1.0 to 0.8 deaths per 1,000 (95% CI: 0.6, 0.9), and inequality between Pardo and White Brazilians from 0.3 to 0.2 per 1,000 (95% CI: 0.1, 0.3).

For peer review only - http://bmjopen.bmj.com/site/about/guidelines.xhtml

Discussion

We simulated the impacts of changes in FHS coverage on mortality in the fifteen largest Brazilian cities using detailed demographic and health data, and estimated relationships at an individual level by cause of death. We found reductions in FHS coverage would lead to higher mortality and exacerbate inequalities among cities. Additionally, marginalized groups including those receiving Bolsa Familia and those of minority race/ethnic groups disproportionately benefit from increasing FHS coverage, and so increased in FHS coverage would also drive reduction in inequalities within cities. We estimated that an increase in FHS primary care coverage by 40 percentage points would still be insufficient on its own to reach SDG target 3.4, as it would only reduce premature mortality from non-communicable diseases by 20% (versus the SDG target of 33%). For SDG target 3.3 of ending deaths from communicable diseases, the result implies that even an increase in FHS primary care coverage by 40 percentage points would be expected to reduce mortality from communicable diseases by 15% but still be far from the SDG of ending deaths from communicable diseases. By contrast, Brazilian cities had already reached SDG target 3.2 of reducing U5MR to less than 25 per 1,000 live births.[23]

The study findings suggest that, in the context of a health system with devolved decision-making to over 5,000 municipal governments, geographic inequality in mortality in Brazil will be greatly affected by future Federal financing and support of the FHS. Investment in primary care is beneficial for local achievement of SDGs for both non-communicable and communicable diseases and reduces geographic inequality in these outcomes. Nonetheless, localities need to engage with non-health sectors to achieve an elimination of health inequalities and should consider setting bolder local targets for accelerating under-5 mortality rates beyond those prescribed in the SDGs.

This is particularly important considering the current economic crisis due to the COVID-19 pandemic in Brazil, which is already responsible for a dramatic increase in poverty and unemployment and will have long-term effects on the most vulnerable groups of the population.[24] Our findings are consistent with previous studies which have shown a synergistic impact of FHS with Bolsa Familia on child mortality,[25,26] and a combined mitigation effect on the increase of premature mortality during the past Brazilian economic recession.[27] Other simulation studies, performed at the aggregate-level, have also indicated how a combined

For peer review only - http://bmjopen.bmj.com/site/about/guidelines.xhtml

expansion of Bolsa Familia and FHS during periods of economic crisis are able to reduce the number of childhood deaths.[12] While our study is focused on Brazilian cities, recent literature has also demonstrated the effectiveness of interventions to expand the primary care coverage to rural areas, as the Mais Medicos Program, on the reduction of premature mortality,[28] and have shown how the end or weakening of such intervention could be responsible for a large number of avoidable deaths.[14,29]

Brazil is one of the few low and middle-income countries with a universal healthcare system, the Unified Health System (SUS), based on one of the world`s largest primary health care programs, the FHS. The expansion of the SUS and FHS during the last 30 years was responsible for large reductions on mortality and health inequalities,[30] but is currently under threat by aggressive and long-term fiscal austerity measures that could undermine its consolidation and even reduce its dimension and effectiveness,[13,14] so robust evidence on the impact of its components - including FHS, are urgently needed.

Our study has a number of strengths and limitations. Our simulation model used estimates of FHS impact on adult mortality based on a unique, individual-level dataset with a rich set of covariates including key socioeconomic and health variables that relate to mortality; nevertheless, factors other than those we have controlled for may be additionally important to consider and may serve as unmeasured factors influencing our results. Additionally this dataset was restricted to the city of Rio de Janeiro where FHS impacts may differ from those in the other major cities included in our study. Indeed the FHS in Rio has major investments in clinics and equipment, a residency programme, and higher salaries for doctors. Our simulation of infant mortality and under-5 mortality was based on hazard ratios from the literature rather than from detailed individual-level data, due to limitations in data availability from the primary datasets we used.

In future research, we plan to examine how rural populations respond differently to urban populations to FHS primary care coverage expansion, and identify factors that may help enhance the effectiveness of FHS in reducing mortality across Brazil. Our work also suggests future research should include more comparative analysis of the health impacts delivered by different models of primary care in Latin America and worldwide. In the meantime, our results suggest that increasing primary care coverage may be critically beneficial to achieving SDG targets

For peer review only - http://bmjopen.bmj.com/site/about/guidelines.xhtml

including reducing within-country inequalities between geographical areas, income and race/ethnic groups in Brazil.

For peer review only

For peer review only - http://bmjopen.bmj.com/site/about/guidelines.xhtml

Competing interests

None.

Author contributions

CM and DR conceived of the study. VS, AT, and BD collected data. SB, TVH, and DR performed the analysis. SB wrote the draft. All authors contributed to editing of the manuscript.

Data sharing

Technical appendix, statistical code, and dataset available from https://github.com/sanjaybasu concurrent with publication.

For peer review only

For peer review only - http://bmjopen.bmj.com/site/about/guidelines.xhtml

References

1 Hone T, Rasella D, Barreto M, Atun R, Majeed A, Millett C. Large Reductions In Amenable Mortality Associated With Brazil's Primary Care Expansion And Strong Health Governance. Health Affairs. 2017; **36**: 149–58.

2 Hone T, Rasella D, Barreto ML, Majeed A, Millett C. Association between expansion of primary healthcare and racial inequalities in mortality amenable to primary care in Brazil: A national longitudinal analysis. PLOS Medicine. 2017; **14**: e1002306.

3 Shi L, Macinko J, Starfield B, Wulu J, Regan J, Politzer R. The relationship between primary care, income inequality, and mortality in US States, 1980-1995. *J Am Board Fam Pract* 2003; **16**: 412–22.

4 Shi L, Macinko J, Starfield B, Politzer R, Xu J. Primary care, race, and mortality in US states. Social Science & Medicine. 2005; **61**: 65–75.

5 Institute of Medicine, Board on Population Health and Public Health Practice, Committee on Integrating Primary Care and Public Health. Primary Care and Public Health: Exploring Integration to Improve Population Health. National Academies Press, 2012 https://books.google.com/books/about/Primary_Care_and_Public_Health.html?hl=&id=qt1dP5ia6vwC.

6 Basu S, Berkowitz SA, Phillips RL, Bitton A, Landon BE, Phillips RS. Association of Primary Care Physician Supply With Population Mortality in the United States, 2005-2015. *JAMA Intern Med* 2019; **179**: 506–14.

7 Rasella D, Harhay MO, Pamponet ML, Aquino R, Barreto ML. Impact of primary health care on mortality from heart and cerebrovascular diseases in Brazil: a nationwide analysis of longitudinal data. *BMJ* 2014; **349**: g4014.

8 Hone T, Macinko J, Millett C. Revisiting Alma-Ata: what is the role of primary health care in achieving the Sustainable Development Goals? The Lancet. 2018; **392**: 1461–72.

9 Pettigrew LM, De Maeseneer J, Anderson M-IP, Essuman A, Kidd MR, Haines A. Primary health care and the Sustainable Development Goals. The Lancet. 2015; **386**: 2119–21.

10 Nilsson M, Griggs D, Visbeck M. Policy: Map the interactions between Sustainable Development Goals. 2016; published online June 15. DOI:10.1038/534320a.

11 Siddiqi S, Aftab W, Siddiqui FJ, *et al.* Global strategies and local implementation of health and health-related SDGs: lessons from consultation in countries across five regions. *BMJ Global Health* 2020; **5**: e002859.

12 Rasella D, Basu S, Hone T, Paes-Sousa R, Ocké-Reis CO, Millett C. Child morbidity and mortality associated with alternative policy responses to the economic crisis in Brazil: A

For peer review only

For peer review only - http://bmjopen.bmj.com/site/about/guidelines.xhtml

nationwide microsimulation study. PLOS Medicine. 2018; **15**: e1002570.

13 Souza LEPF de, de Souza LEPF, de Barros RD, *et al.* The potential impact of austerity on attainment of the Sustainable Development Goals in Brazil. BMJ Global Health. 2019; **4**: e001661.

14 Rasella D, Hone T, de Souza LE, Tasca R, Basu S, Millett C. Mortality associated with alternative primary healthcare policies: a nationwide microsimulation modelling study in Brazil. *BMC Med* 2019; **17**: 82.

15 Dalby S, Horton S, Mahon R. Global governance challenges in achieving the Sustainable Development Goals. Achieving the Sustainable Development Goals. 2019; : 1–18.

16 Informacio e Gestao da Atencao Basica. Cobertua da Atencao Basica. https://egestorab.saude.gov.br/paginas/acessoPublico/relatorios/relHistoricoCoberturaAB.xhtml (accessed Nov 28, 2020).

17 Basu S. Microsimulation. Oxford Medicine Online. 2017. DOI:10.1093/med/9780190667924.003.0008.

18 Hone T, Saraceni V, Medina Coeli C, *et al.* Primary healthcare expansion and mortality in Brazil's urban poor: A cohort analysis of 1.2 million adults. *PLoS Med* 2020; **17**: e1003357.

19 Brazil's Cadastro Unico: an enduring bridge to consolidated targeting efforts. https://socialprotection.org/discover/publications/brazil%E2%80%99s-cadastro-unico-enduring-bridge-consolidated-targeting-efforts (accessed Sept 30, 2020).

20 WHO | International Classification of Diseases (ICD) Information Sheet. 2019; published online Oct 11. http://www.who.int/classifications/icd/factsheet/en/ (accessed Sept 30, 2020).

21 Bastos ML, Menzies D, Hone T, Dehghani K, Trajman A. The impact of the Brazilian family health on selected primary care sensitive conditions: A systematic review. *PLoS One* 2017; **12**: e0182336.

22 IBGE. https://www.ibge.gov.br/ (accessed Nov 29, 2020).

23 Datasus. DATASUS. http://www2.datasus.gov.br/DATASUS/index.php?area=0205 (accessed Nov 29, 2020).

24 Marson FAL, Ortega MM. COVID-19 in Brazil. *Pulmonology* 2020; **26**: 241–4.

25 Guanais FC. The Combined Effects of the Expansion of Primary Health Care and Conditional Cash Transfers on Infant Mortality in Brazil, 1998–2010. *Am J Public Health* 2015; **105**: S593–9.

26 Rasella D, Aquino R, Santos CAT, Paes-Sousa R, Barreto ML. Effect of a conditional cash

For peer review only - http://bmjopen.bmj.com/site/about/guidelines.xhtml

transfer programme on childhood mortality: a nationwide analysis of Brazilian municipalities. *Lancet* 2013; **382**: 57–64.

27 Hone T, Mirelman AJ, Rasella D, *et al.* Effect of economic recession and impact of health and social protection expenditures on adult mortality: a longitudinal analysis of 5565 Brazilian municipalities. *Lancet Glob Health* 2019; **7**: e1575–83.

28 Hone T, Powell-Jackson T, Santos LMP, *et al.* Impact of the Programa Mais médicos (more doctors Programme) on primary care doctor supply and amenable mortality: quasi-experimental study of 5565 Brazilian municipalities. *BMC Health Serv Res* 2020; **20**: 873.

29 Francesconi GV, Tasca R, Basu S, Rocha TAH, Rasella D. Mortality associated with alternative policy options for primary care and the Mais Médicos (More Doctors) Program in Brazil: forecasting future scenarios. *Rev Panam Salud Publica* 2020; **44**: e31.

30 Castro MC, Massuda A, Almeida G, *et al.* Brazil's unified health system: the first 30 years and prospects for the future. *Lancet* 2019; **394**: 345–56.

For peer review only

For peer review only - http://bmjopen.bmj.com/site/about/guidelines.xhtml

Declaration of Interests

None

Acknowledgements

This work was supported by a grant from the Health Systems Research Initiative with funding from the Foreign, Commonwealth and Development Office, the UK Medical Research Council and Wellcome, in collaboration with the UK Economic and Social Research Council (grant no. MR/P014593/1).

For peer review only

For peer review only - http://bmjopen.bmj.com/site/about/guidelines.xhtml

Figure Legends

Figure 1: Projected variations in (A) all-cause crude mortality, (B) all-cause age-standardized mortality, (C) infant mortality and (D) under-5 mortality given different levels of Family Health Strategy (FHS) program primary care coverage. See Table 1 for current coverage levels corresponding to a 0% change on the x-axis. See 95% confidence intervals in Supporting Information Figure 2.

Figure 2: Projected changes in all-cause and cause-specific age-standardized mortality given (A) a 20 percentage point decline or (B) a 40 percentage point increase in Family Health Strategy (FHS) program primary care coverage from the baseline levels indicated in Table 1. Legend: BF: participation in the Bolsa Familia program.

For peer review only

For peer review only - http://bmjopen.bmj.com/site/about/guidelines.xhtml

Tables and Figures

Table 1: Demographics, primary care coverage, and mortality among the fifteen largest Brazilian cities. FHS: Estratégia de Saúde da Família (Family Health Strategy) primary care program (2016).[19]

City	Age, less than 15 years, %	Age, greater than 64 years, %	Female, %	Race, % White	Race, % Black	Ethnicity, % Pardo	Income, % below poverty line	Education, % graduated secondary school (age 18-20)	FHS primary care coverage, %	Mortality, all cause, crude (per 1,000)	Mortality, all cause, age adjusted (per 1,000)	Infant mortality (per 1,000 live births)	Under-5 mortality (per 1,000 live births)
Belo Horizonte	29.9	4.7	52.7	40.5	12.6	46.5	5.6	47.5	76.5	5.76	4.95	13.0	15.2
Belém	24.8	5.5	52.1	21.2	9.1	69.3	14.9	37.1	23.5	5.49	6.17	16.1	17.2
Brasília	23.7	5.0	52.2	40.0	10.6	48.3	4.9	53.5	37.8	3.93	4.98	14.0	15.8
Campinas	19.3	5.8	51.8	57.2	8.9	30.8	3.2	53.2	44.5	5.60	4.86	11.8	13.7
Curitiba	20.0	4.9	52.3	74.3	3.8	58.5	1.7	57.8	36.4	5.19	4.86	11.9	13.6
Fortaleza	22.6	4.9	53.2	32.3	5.8	61.4	12.1	45.4	45.9	4.93	5.40	15.8	16.9
Goiânia	20.8	4.2	52.3	43.2	6.4	49.8	3.1	57.0	44.3	5.42	5.99	13.1	15.0
Maceió	25.0	4.4	53.2	27.1	5.6	66.7	15.6	42.6	29.0	5.91	7.04	22.0	24.0
Manaus	28.2	3.2	51.2	20.1	2.3	77.0	12.9	38.8	27.0	4.34	6.84	14.2	15.2
Porto Alegre	18.8	6.4	53.6	78.0	11.4	10.2	3.8	48.2	55.0	7.42	5.43	11.6	13.1
Recife	20.9	5.5	53.8	36.9	9.1	52.7	13.2	46.7	54.8	6.43	6.05	15.6	12.5
Rio de Janeiro	19.4	7.6	53.2	48.5	11.6	39.2	5.0	45.9	62.9	8.14	5.97	13.0	14.6
Salvador	20.7	4.3	53.3	14.8	38.0	46.8	11.4	41.8	26.7	5.32	5.90	14.9	12.0
São Luís	23.7	3.8	53.2	19.9	15.5	63.9	13.8	53.1	34.4	4.64	5.91	18.1	19.8
São Paulo	20.8	5.5	52.6	57.9	8.7	30.4	4.3	50.5	35.4	5.71	5.09	13.2	14.7

For peer review only - http://bmjopen.bmj.com/site/about/guidelines.xhtml

Table 2: Relative impact on cause-specific mortality given changes in the percentage point coverage in the FHS primary care strategy (Estratégia de Saúde da Família). The cells show the ratio of mortality by cause under different levels of FHS coverage, compared to the current mortality rate (at 0% change in FHS coverage), the reference column. 95% confidence intervals in Supporting Information Table 2.

	**Ratio of mortality by cause, compared to current mortality rate (at 0%)**						
**Percentage point change in FHS coverage:**	**-20%**	**-10%**	**0%**	**10%**	**20%**	**30%**	**40%**
All causes	1.11	1.05	1.00	0.95	0.89	0.84	0.78
Infections (excluding HIV, TB, malaria, NTDs)	1.11	1.05	1.00	0.95	0.89	0.84	0.79
HIV	1.08	1.04	1.00	0.96	0.92	0.88	0.84
TB, malaria, NTDs	1.03	1.02	1.00	0.98	0.97	0.95	0.94
Respiratory	1.10	1.05	1.00	0.95	0.90	0.85	0.80
Nutrition	1.08	1.04	1.00	0.96	0.92	0.87	0.83
Neoplasms	1.11	1.05	1.00	0.95	0.89	0.84	0.78
Nervous system	1.06	1.03	1.00	0.97	0.94	0.92	0.89
Endocrine	1.11	1.05	1.00	0.95	0.89	0.84	0.79
Mental/substance use	1.10	1.05	1.00	0.95	0.90	0.84	0.79
Stroke	1.14	1.07	1.00	0.93	0.86	0.80	0.73
Heart disease	1.11	1.05	1.00	0.95	0.89	0.84	0.79
Other cardiovascular	1.13	1.06	1.00	0.94	0.87	0.81	0.74
Digestive	1.10	1.05	1.00	0.95	0.90	0.84	0.79

For peer review only - http://bmjopen.bmj.com/site/about/guidelines.xhtml

Genitourinary	1.10	1.05	1.00	0.95	0.90	0.86	0.81
Unintentional injuries	1.13	1.07	1.00	0.93	0.87	0.80	0.74
Intentional injuries	1.16	1.08	1.00	0.92	0.84	0.75	0.67
Maternal	1.01	1.00	1.00	1.00	0.99	0.99	0.99

For peer review only

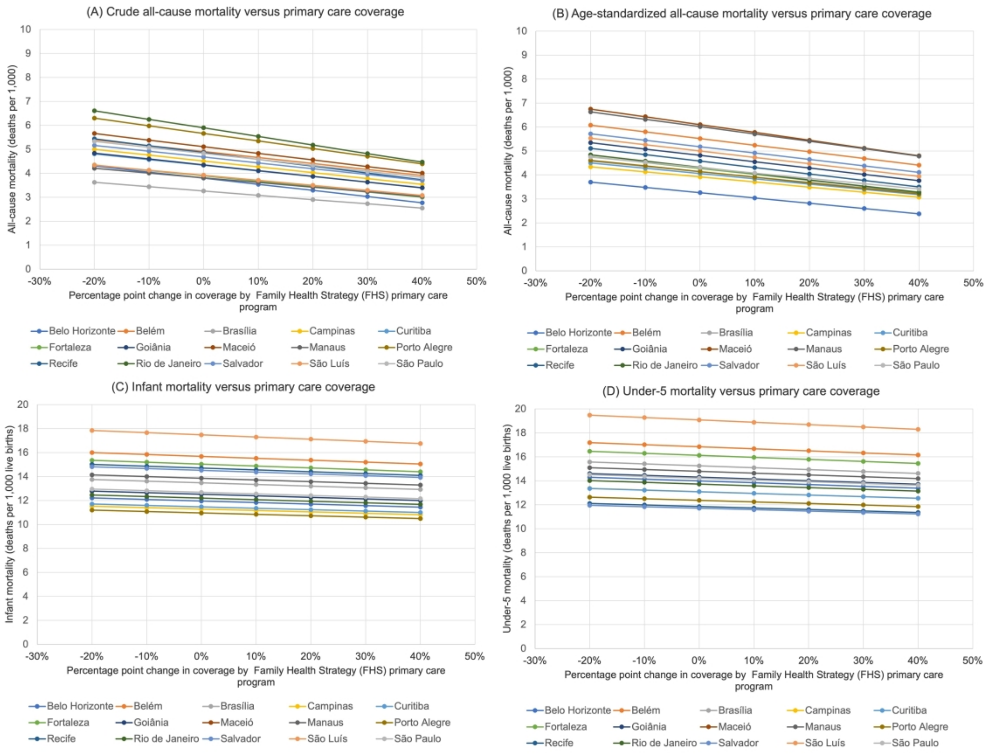

FIGURE 1

417x315mm (300 x 300 DPI)

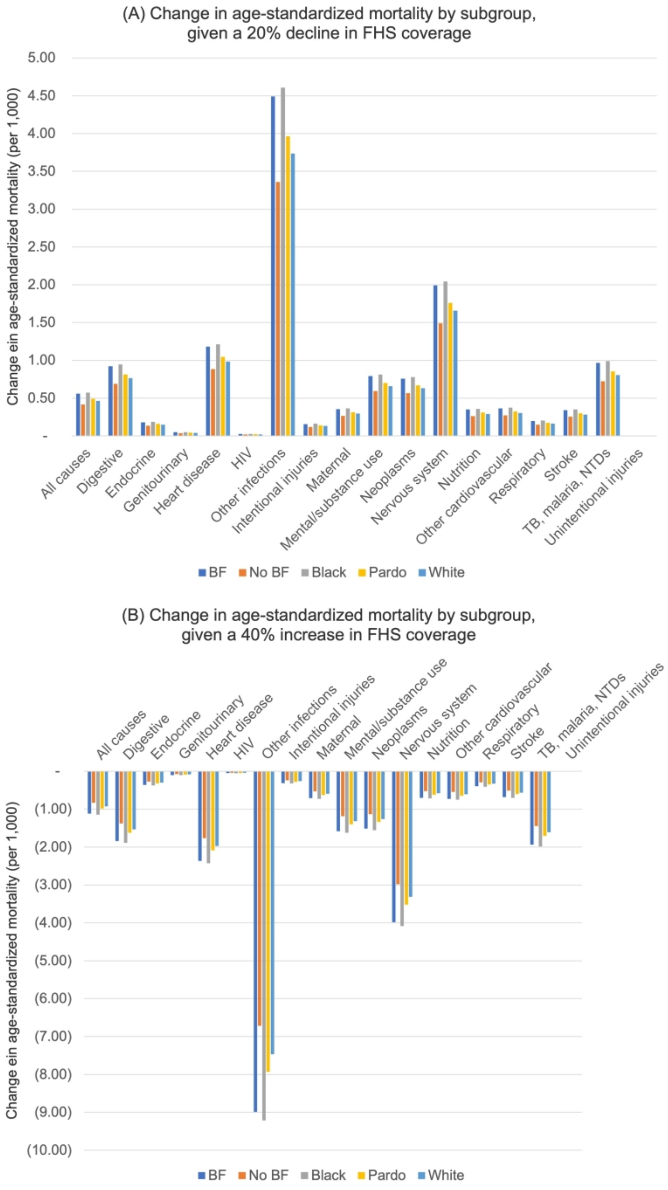

FIGURE 2

203x357mm (300 x 300 DPI)

Supporting Information

For peer review only

For peer review only - http://bmjopen.bmj.com/site/about/guidelines.xhtml

Supporting Information Table 1: Hazard ratios for death by cause group, subdivided by sex, race/ethnicity, and whether or not the family receives Bolsa Familia (BF) benefits.

Cause	Subgroup	Hazard Ratio	Lower 95% Confidence interval	Upper 95% Confidence interval
All Causes	Males	0.68	0.64	0.71
All Causes	Females	0.49	0.47	0.52
All Causes	Race White	0.60	0.56	0.64
All Causes	Race Black	0.50	0.47	0.54
All Causes	Race Pardo	0.57	0.54	0.60
All Causes	No Bf	0.64	0.61	0.67
All Causes	Bf	0.52	0.49	0.54
Infectious And Parasitic Diseases	Males	0.82	0.62	1.08
Infectious And Parasitic Diseases	Females	0.53	0.41	0.67
Infectious And Parasitic Diseases	Race White	0.86	0.63	1.17
Infectious And Parasitic Diseases	Race Black	0.68	0.45	1.02
Infectious And Parasitic Diseases	Race Pardo	0.52	0.39	0.69
Infectious And Parasitic Diseases	No Bf	0.68	0.52	0.88
Infectious And Parasitic Diseases	Bf	0.63	0.49	0.82
Hiv/Aids	Males	1.15	0.88	1.52
Hiv/Aids	Females	0.68	0.55	0.83
Hiv/Aids	Race White	0.67	0.44	1.01
Hiv/Aids	Race Black	0.69	0.51	0.93
Hiv/Aids	Race Pardo	1.02	0.80	1.31
Hiv/Aids	No Bf	1.20	0.86	1.68
Hiv/Aids	Bf	0.73	0.61	0.89

For peer review only

For peer review only - http://bmjopen.bmj.com/site/about/guidelines.xhtml

Tuberculosis, Malaria, And Ntds	Males	0.91	0.61	1.35
Tuberculosis, Malaria, And Ntds	Females	0.24	0.13	0.43
Tuberculosis, Malaria, And Ntds	Race White	0.61	0.29	1.28
Tuberculosis, Malaria, And Ntds	Race Black	0.49	0.25	0.95
Tuberculosis, Malaria, And Ntds	Race Pardo	0.67	0.42	1.06
Tuberculosis, Malaria, And Ntds	No Bf	0.68	0.38	1.22
Tuberculosis, Malaria, And Ntds	Bf	0.56	0.38	0.82
Maternal Causes (Females Only)	Females	0.97	0.60	1.56
Maternal Causes (Females Only)	Race White	1.01	0.46	2.23
Maternal Causes (Females Only)	Race Black	1.09	0.46	2.56
Maternal Causes (Females Only)	Race Pardo	0.82	0.37	1.82
Maternal Causes (Females Only)	No Bf	1.93	0.63	5.90
Maternal Causes (Females Only)	Bf	0.81	0.48	1.39
Nutritional Deficiencies	Males	0.59	0.26	1.33
Nutritional Deficiencies	Females	0.52	0.28	0.98
Nutritional Deficiencies	Race White	0.21	0.05	0.97
Nutritional Deficiencies	Race Black	0.40	0.14	1.19
Nutritional Deficiencies	Race Pardo	0.92	0.50	1.69
Nutritional Deficiencies	No Bf	0.99	0.53	1.88
Nutritional Deficiencies	Bf	0.23	0.10	0.54
Diseases Of The Nervous System	Males	0.52	0.28	0.98
Diseases Of The Nervous System	Females	0.58	0.38	0.91
Diseases Of The Nervous System	Race White	0.46	0.25	0.88

For peer review only

For peer review only - http://bmjopen.bmj.com/site/about/guidelines.xhtml

Diseases Of The Nervous System	Race Black	0.83	0.40	1.75
Diseases Of The Nervous System	Race Pardo	0.55	0.31	0.97
Diseases Of The Nervous System	No Bf	0.48	0.29	0.82
Diseases Of The Nervous System	Bf	0.68	0.41	1.12
Endocrine Disorders	Males	0.74	0.57	0.95
Endocrine Disorders	Females	0.50	0.41	0.61
Endocrine Disorders	Race White	0.58	0.43	0.79
Endocrine Disorders	Race Black	0.55	0.40	0.74
Endocrine Disorders	Race Pardo	0.60	0.47	0.76
Endocrine Disorders	No Bf	0.59	0.47	0.75
Endocrine Disorders	Bf	0.59	0.47	0.73
Mental And Substance Use Disorders	Males	0.66	0.41	1.05
Mental And Substance Use Disorders	Females	0.27	0.15	0.50
Mental And Substance Use Disorders	Race White	0.67	0.32	1.41
Mental And Substance Use Disorders	Race Black	0.17	0.05	0.56
Mental And Substance Use Disorders	Race Pardo	0.53	0.32	0.86
Mental And Substance Use Disorders	No Bf	0.75	0.36	1.54
Mental And Substance Use Disorders	Bf	0.40	0.26	0.61
Stroke	Males	0.77	0.63	0.94
Stroke	Females	0.45	0.38	0.53
Stroke	Race White	0.67	0.53	0.85
Stroke	Race Black	0.45	0.34	0.58
Stroke	Race Pardo	0.60	0.50	0.73

For peer review only

For peer review only - http://bmjopen.bmj.com/site/about/guidelines.xhtml

Stroke	No Bf	0.65	0.54	0.78
Stroke	Bf	0.50	0.42	0.60
Heart Disease	Males	0.59	0.52	0.67
Heart Disease	Females	0.41	0.37	0.46
Heart Disease	Race White	0.47	0.40	0.56
Heart Disease	Race Black	0.43	0.35	0.52
Heart Disease	Race Pardo	0.51	0.44	0.58
Heart Disease	No Bf	0.52	0.46	0.59
Heart Disease	Bf	0.47	0.42	0.54
Other Cardiovascular Diseases	Males	0.68	0.54	0.86
Other Cardiovascular Diseases	Females	0.49	0.41	0.60
Other Cardiovascular Diseases	Race White	0.63	0.47	0.84
Other Cardiovascular Diseases	Race Black	0.41	0.30	0.56
Other Cardiovascular Diseases	Race Pardo	0.65	0.52	0.81
Other Cardiovascular Diseases	No Bf	0.64	0.52	0.79
Other Cardiovascular Diseases	Bf	0.50	0.40	0.62
Digestive Diseases	Males	0.67	0.53	0.84
Digestive Diseases	Females	0.52	0.41	0.66
Digestive Diseases	Race White	0.61	0.46	0.83
Digestive Diseases	Race Black	0.47	0.32	0.70
Digestive Diseases	Race Pardo	0.61	0.48	0.79
Digestive Diseases	No Bf	0.61	0.47	0.79
Digestive Diseases	Bf	0.59	0.48	0.74
Genitourinary Diseases	Males	0.67	0.51	0.88

For peer review only

For peer review only - http://bmjopen.bmj.com/site/about/guidelines.xhtml

Genitourinary Diseases	Females	0.52	0.42	0.65
Genitourinary Diseases	Race White	0.63	0.46	0.87
Genitourinary Diseases	Race Black	0.60	0.43	0.83
Genitourinary Diseases	Race Pardo	0.57	0.44	0.74
Genitourinary Diseases	No Bf	0.64	0.51	0.80
Genitourinary Diseases	Bf	0.52	0.40	0.68
Unintentional Injuries	Males	0.56	0.43	0.74
Unintentional Injuries	Females	0.43	0.32	0.58
Unintentional Injuries	Race White	0.50	0.33	0.76
Unintentional Injuries	Race Black	0.47	0.31	0.71
Unintentional Injuries	Race Pardo	0.53	0.40	0.71
Unintentional Injuries	No Bf	0.55	0.40	0.78
Unintentional Injuries	Bf	0.46	0.36	0.60
Intentional Injuries	Males	0.42	0.33	0.53
Intentional Injuries	Females	0.30	0.18	0.50
Intentional Injuries	Race White	0.51	0.33	0.79
Intentional Injuries	Race Black	0.45	0.27	0.73
Intentional Injuries	Race Pardo	0.34	0.24	0.46
Intentional Injuries	No Bf	0.72	0.51	1.01
Intentional Injuries	Bf	0.28	0.21	0.37
Neoplasms	Males	0.97	0.86	1.11
Neoplasms	Females	0.61	0.55	0.68
Neoplasms	Race White	0.68	0.59	0.78
Neoplasms	Race Black	0.69	0.57	0.84
Neoplasms	Race Pardo	0.83	0.74	0.93

For peer review only - http://bmjopen.bmj.com/site/about/guidelines.xhtml
For peer review only

Neoplasms	No Bf	0.77	0.69	0.86
Neoplasms	Bf	0.73	0.65	0.82
Respiratory Infections And Diseases	Males	0.84	0.73	0.98
Respiratory Infections And Diseases	Females	0.49	0.42	0.56
Respiratory Infections And Diseases	Race White	0.73	0.62	0.87
Respiratory Infections And Diseases	Race Black	0.57	0.44	0.72
Respiratory Infections And Diseases	Race Pardo	0.58	0.49	0.69
Respiratory Infections And Diseases	No Bf	0.68	0.59	0.79
Respiratory Infections And Diseases	Bf	0.61	0.52	0.70

For peer review only

For peer review only - http://bmjopen.bmj.com/site/about/guidelines.xhtml

Supporting Information Table 2: (A) 95% lower confidence interval estimates and (B) 95% upper confidence interval estimates around the relative impact on cause-specific mortality given changes in the percentage point coverage in the FHS program (Estratégia de Saúde da Família) primary care program. The cells show the ratio of mortality by cause under different levels of FHS coverage, compared to the current mortality rate (at 0% change in FHS coverage), the reference column.

For peer review only

For peer review only - http://bmjopen.bmj.com/site/about/guidelines.xhtml

(A)

	Ratio of mortality by cause, compared to current mortality rate (at 0%)						
**Percentage point change in FHS coverage:**	**-20%**	**-10%**	**0%**	**10%**	**20%**	**30%**	**40%**
All causes	1.07	1.00	0.93	0.86	0.79	0.72	0.65
Infections (excluding HIV, TB, malaria, NTDs)	1.07	1.00	0.93	0.86	0.79	0.72	0.66
HIV	1.04	0.99	0.93	0.88	0.82	0.76	0.70
TB, malaria, NTDs	1.00	0.97	0.93	0.90	0.86	0.82	0.78
Respiratory	1.06	1.00	0.93	0.87	0.80	0.73	0.67
Nutrition	1.05	0.99	0.93	0.87	0.81	0.75	0.69
Neoplasms	1.07	1.00	0.93	0.86	0.79	0.72	0.65
Nervous system	1.02	0.98	0.93	0.89	0.84	0.79	0.74
Endocrine	1.07	1.00	0.93	0.86	0.79	0.72	0.65
Mental/substance use	1.07	1.00	0.93	0.86	0.80	0.73	0.66
Stroke	1.10	1.01	0.93	0.85	0.77	0.69	0.61
Heart disease	1.07	1.00	0.93	0.86	0.80	0.73	0.66
Other cardiovascular	1.09	1.01	0.93	0.85	0.77	0.70	0.62
Digestive	1.07	1.00	0.93	0.86	0.80	0.73	0.66
Genitourinary	1.06	1.00	0.93	0.87	0.80	0.74	0.67
Unintentional injuries	1.09	1.01	0.93	0.85	0.77	0.69	0.62

For peer review only - http://bmjopen.bmj.com/site/about/guidelines.xhtml

Intentional injuries	1.13	1.03	0.93	0.84	0.74	0.65	0.56
Maternal	0.97	0.95	0.93	0.91	0.88	0.85	0.82

For peer review only

For peer review only - http://bmjopen.bmj.com/site/about/guidelines.xhtml

(B)

	Ratio of mortality by cause, compared to current mortality rate (at 0%)						
**Percentage point change in FHS coverage:**	**-20%**	**-10%**	**0%**	**10%**	**20%**	**30%**	**40%**
All causes	1<br>.15	1<br>.11	1<br>.07	1<br>.03	0<br>.99	0<br>.95	0<br>.91
Infections (excluding HIV, TB, malaria, NTDs)	1<br>.14	1<br>.11	1<br>.07	1<br>.03	0<br>.99	0<br>.96	0<br>.92
HIV	1<br>.11	1<br>.09	1<br>.07	1<br>.05	1<br>.02	1<br>.00	0<br>.98
TB, malaria, NTDs	1<br>.07	1<br>.07	1<br>.07	1<br>.07	1<br>.08	1<br>.08	1<br>.09
Respiratory	1<br>.14	1<br>.10	1<br>.07	1<br>.03	1<br>.00	0<br>.97	0<br>.94
Nutrition	1<br>.12	1<br>.09	1<br>.07	1<br>.04	1<br>.02	0<br>.99	0<br>.97
Neoplasms	1<br>.14	1<br>.11	1<br>.07	1<br>.03	0<br>.99	0<br>.95	0<br>.91
Nervous system	1<br>.09	1<br>.08	1<br>.07	1<br>.06	1<br>.05	1<br>.04	1<br>.04
Endocrine	1<br>.14	1<br>.11	1<br>.07	1<br>.03	0<br>.99	0<br>.96	0<br>.92
Mental/substance use	1<br>.14	1<br>.10	1<br>.07	1<br>.03	1<br>.00	0<br>.96	0<br>.93
Stroke	1<br>.17	1<br>.12	1<br>.07	1<br>.02	0<br>.96	0<br>.91	0<br>.85
Heart disease	1<br>.14	1<br>.11	1<br>.07	1<br>.03	0<br>.99	0<br>.96	0<br>.92
Other cardiovascular	1<br>.17	1<br>.12	1<br>.07	1<br>.02	0<br>.97	0<br>.92	0<br>.86
Digestive	1<br>.14	1<br>.10	1<br>.07	1<br>.03	1<br>.00	0<br>.96	0<br>.92
Genitourinary	1<br>.13	1<br>.10	1<br>.07	1<br>.04	1<br>.00	0<br>.97	0<br>.94
Unintentional injuries	1	1	1	1	0	0	0

For peer review only - http://bmjopen.bmj.com/site/about/guidelines.xhtml

	.17	.12	.07	.02	.97	.92	.86
Intentional injuries	1<br>.20	1<br>.14	1<br>.07	1<br>.00	0<br>.93	0<br>.86	0<br>.78
Maternal	1<br>.04	1<br>.05	1<br>.07	1<br>.09	1<br>.10	1<br>.13	1<br>.15

Supporting Information Figure 1: Hazard ratios for FHS users (compared to non-users) by cause of death.

For peer review only

For peer review only - http://bmjopen.bmj.com/site/about/guidelines.xhtml

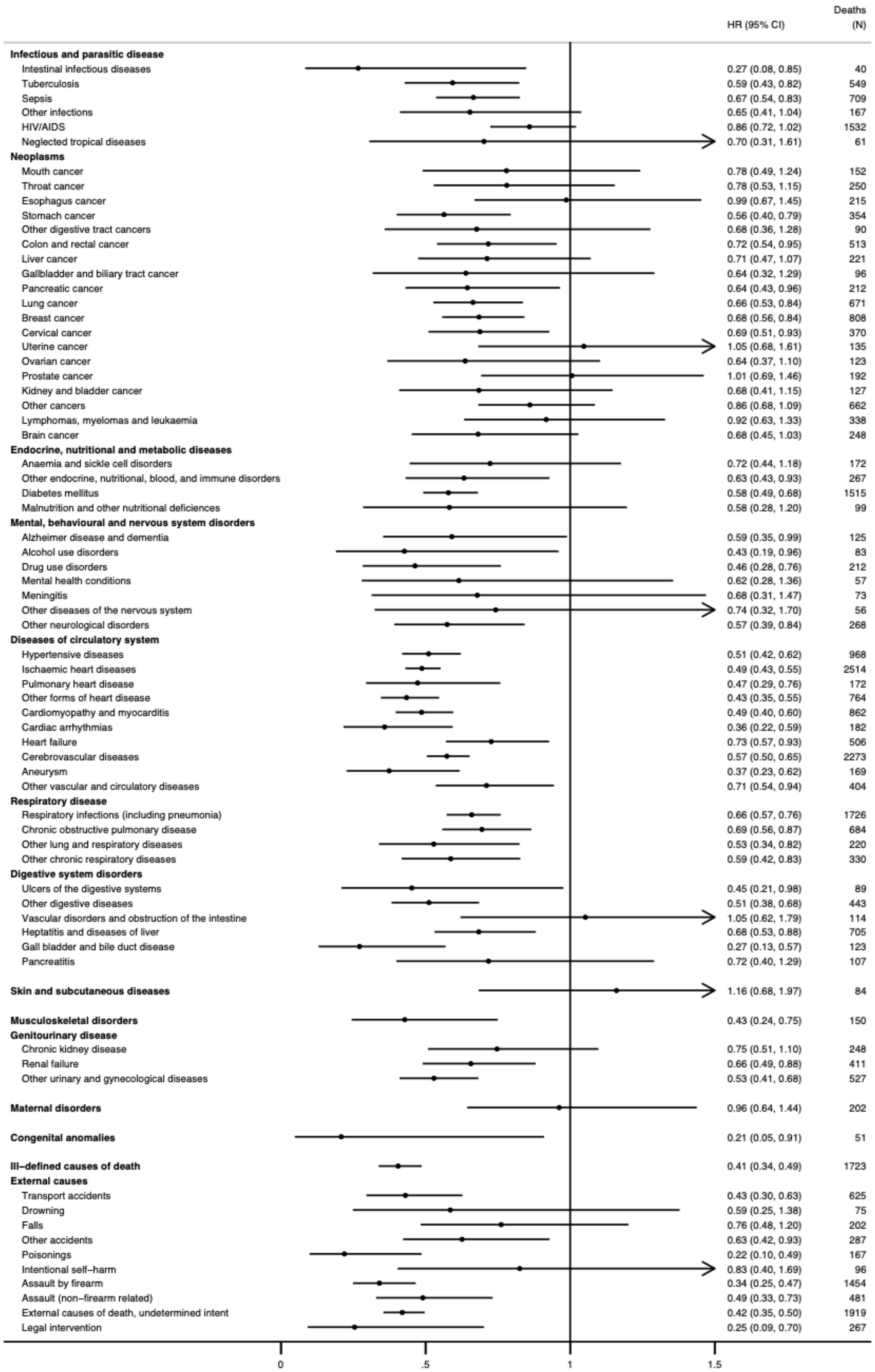

Supporting Information Figure 2: Uncertainty estimates around mean projected variations in (A) all-cause crude mortality, (B) all-cause age-standardized mortality, (C) infant mortality and

For peer review only - http://bmjopen.bmj.com/site/about/guidelines.xhtml

(D) under-5 mortality given different levels of Family Health Strategy (FHS) program primary care coverage. See Table 1 for current coverage levels corresponding to a 0% change on the x-axis. Mean values are population-weighted across all 15 Brazilian cities.

(A)

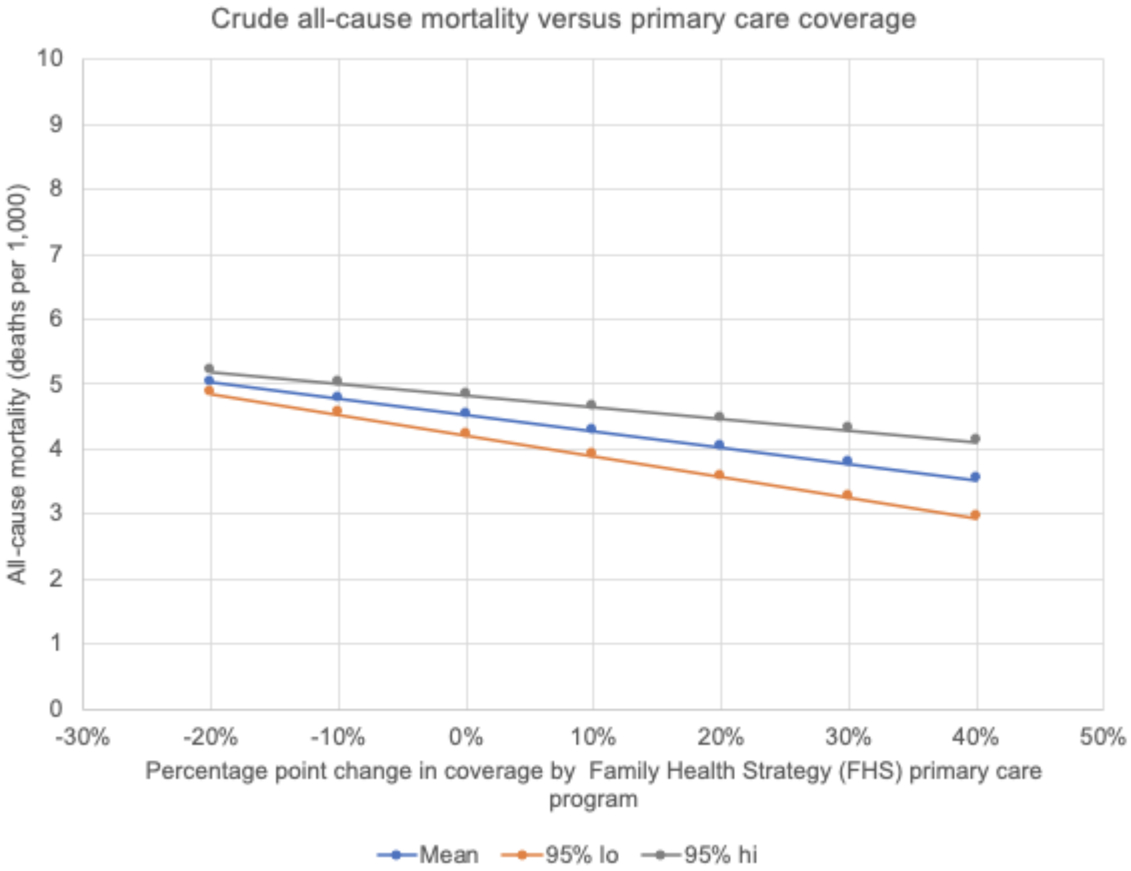

For peer review only - http://bmjopen.bmj.com/site/about/guidelines.xhtml

(B)

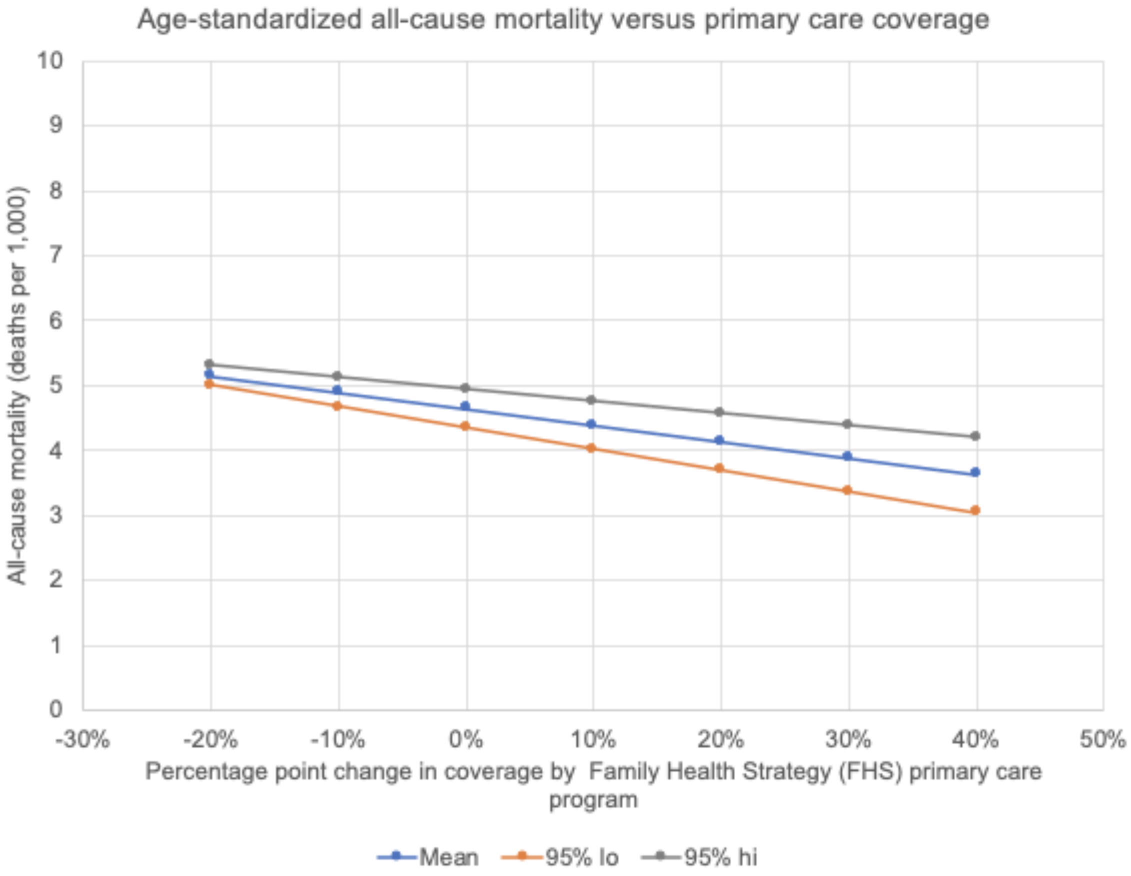

w only

For peer review only - http://bmjopen.bmj.com/site/about/guidelines.xhtml

(C)

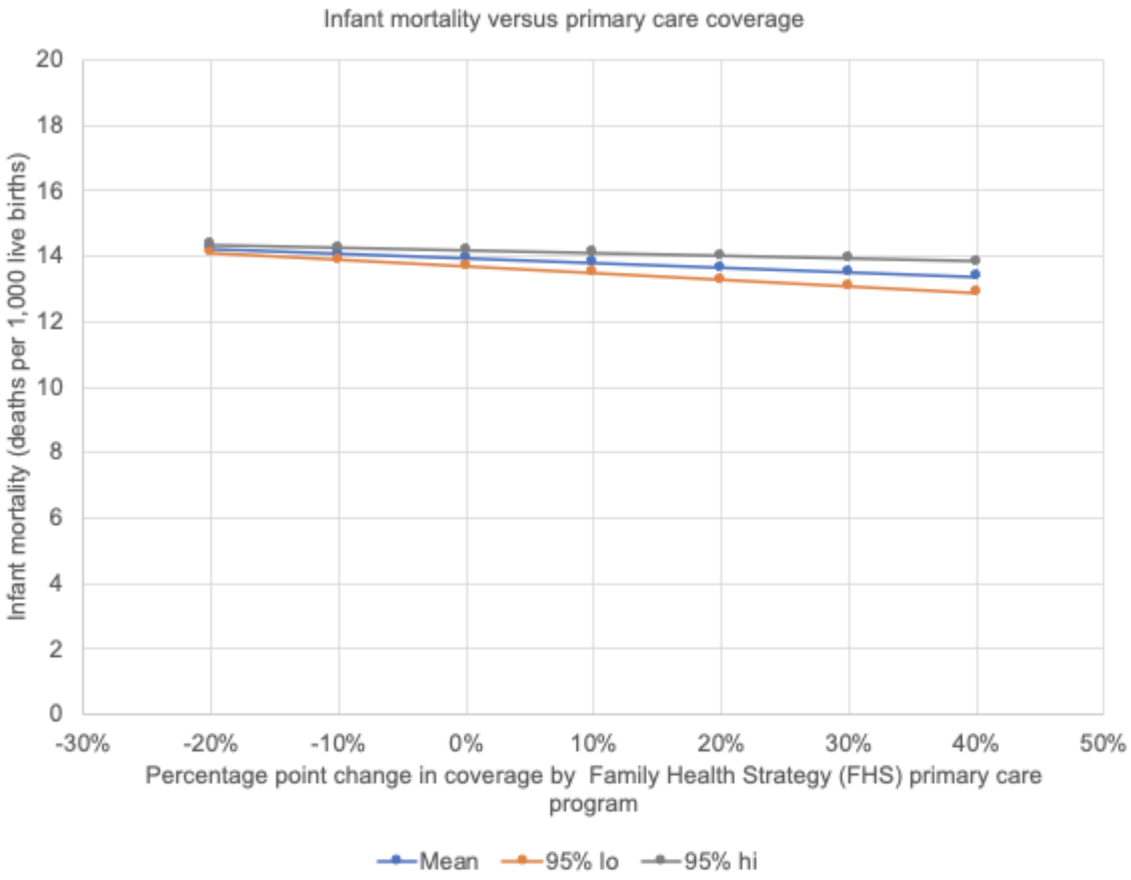

For peer review only - http://bmjopen.bmj.com/site/about/guidelines.xhtml

(D)

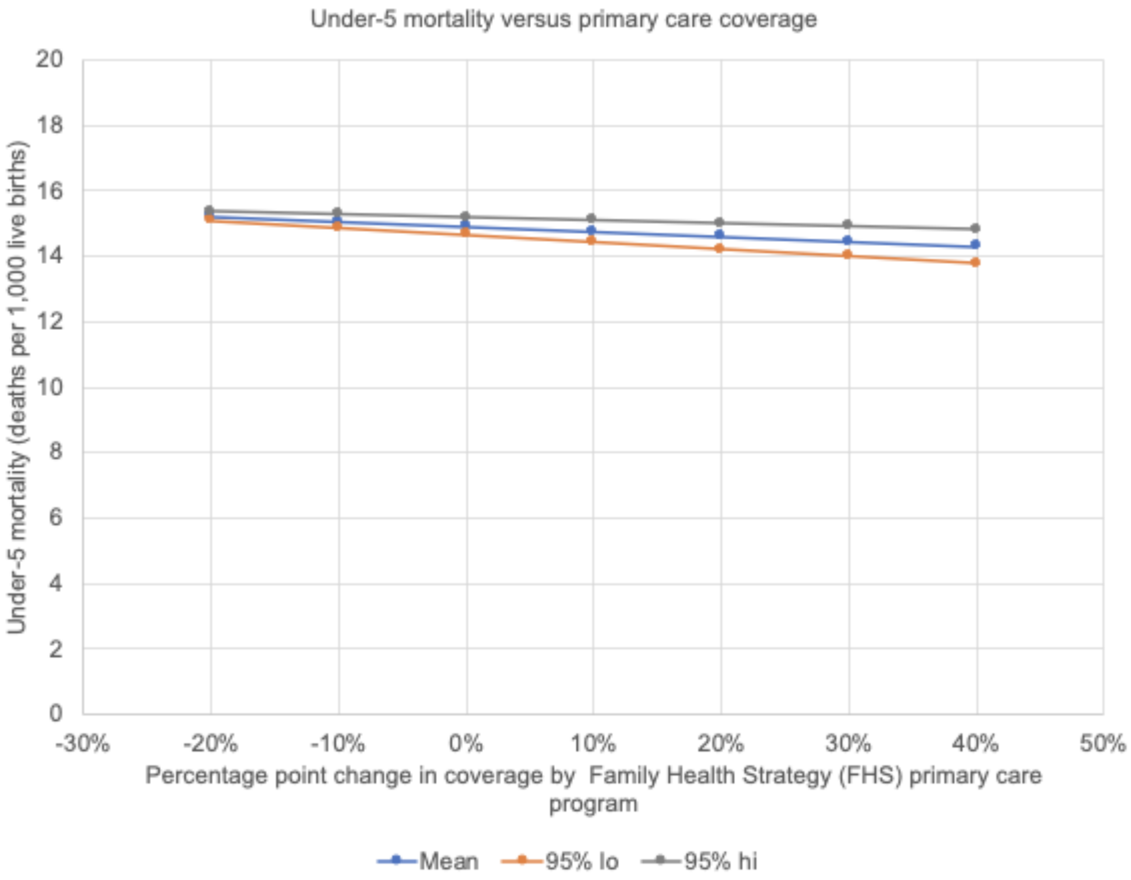

For peer review only - http://bmjopen.bmj.com/site/about/guidelines.xhtml

BMJ Open

The contribution of primary care expansion to Sustainable Development Goal Three for health: A microsimulation of the fifteen largest cities in Brazil

Journal:	*BMJ Open*
Manuscript ID	bmjopen-2021-049251.R1
Article Type:	Original research
Date Submitted by the Author:	24-Aug-2021
Complete List of Authors:	Basu, Sanjay; Harvard Medical School,<br>Hone, Thomas; Imperial College London<br>Villela, Daniel; Oswaldo Cruz Foundation<br>Saraceni, Valeria; FIOCRUZ<br>Trajman, Anete; Universidade Gama Filho<br>Durovni, Betina; Fundação Oswaldo Cruz<br>Millett, Christopher; Imperial College London, Primary Care and Social Medicine<br>Rasella, Davide; Federal University of Bahia
**Primary Subject Heading**:	Health policy
Secondary Subject Heading:	Epidemiology
Keywords:	PRIMARY CARE, PUBLIC HEALTH, GENERAL MEDICINE (see Internal Medicine)

SCHOLARONE™
Manuscripts

BMJ

I, the Submitting Author has the right to grant and does grant on behalf of all authors of the Work (as defined in the below author licence), an exclusive licence and/or a non-exclusive licence for contributions from authors who are: i) UK Crown employees; ii) where BMJ has agreed a CC-BY licence shall apply, and/or iii) in accordance with the terms applicable for US Federal Government officers or employees acting as part of their official duties; on a worldwide, perpetual, irrevocable, royalty-free basis to BMJ Publishing Group Ltd ("BMJ") its licensees and where the relevant Journal is co-owned by BMJ to the co-owners of the Journal, to publish the Work in this journal and any other BMJ products and to exploit all rights, as set out in our licence.

The Submitting Author accepts and understands that any supply made under these terms is made by BMJ to the Submitting Author unless you are acting as an employee on behalf of your employer or a postgraduate student of an affiliated institution which is paying any applicable article publishing charge ("APC") for Open Access articles. Where the Submitting Author wishes to make the Work available on an Open Access basis (and intends to pay the relevant APC), the terms of reuse of such Open Access shall be governed by a Creative Commons licence – details of these licences and which Creative Commons licence will apply to this Work are set out in our licence referred to above.

Other than as permitted in any relevant BMJ Author's Self Archiving Policies, I confirm this Work has not been accepted for publication elsewhere, is not being considered for publication elsewhere and does not duplicate material already published. I confirm all authors consent to publication of this Work and authorise the granting of this licence.

For peer review only

The contribution of primary care expansion to Sustainable Development Goal Three for health: A microsimulation of the fifteen largest cities in Brazil

Sanjay Basu MD PhD[1,2,3,4*], Thomas V. Hone PhD[1], Daniel Villela[5], Valeria Saraceni[6], Anete Trajman[7], Betina Durovni[8], Christopher Millett PhD[1,9,10], Davide Rasella PhD[11,1]

[1] Public Health Policy Evaluation Unit, Imperial College London, London, UK

[2] Center for Primary Care, Harvard Medical School, Boston, MA

[3]Ariadne Labs, Brigham and Women's Hospital and Harvard T.H. Chan School of Public Health, Boston, MA

[4] Institute of Health Policy, Management and Evaluation, University of Toronto, Toronto, Canada

[5] Program of Scientific Computing, Oswaldo Cruz Foundation, Rio de Janeiro, Brazil

[6] Secretaria Municipal de Saúde do Rio de Janeiro, Rio de Janeiro, Brazil

[7] Centro de Estudos Estratégicos, Oswaldo Cruz Foundation, Rio de Janeiro, Brazil

[8] Programa de Pós-graduação em Clínica Médica and Mestrado Profissional em Atenção Primária à Saúde, Federal University of Rio de Janeiro, Brazil

[9] Department of Preventive Medicine, School of Medicine, University of São Paulo, São Paulo, Brazil

[10] Center of Data and Knowledge Integration for Health (CIDACS), Instituto Gonçalo Muniz, Fundação Oswaldo Cruz, Salvador, Brazil

[11] ISGlobal, Hospital Clínic - Universitat de Barcelona, Barcelona, Spain

* to whom correspondence should be addressed

sanjayb493@gmail.com

635 Huntington Avenue

Second Floor

Boston, MA 02115

(617) 432-2222

Word count: 3,304/4,000

For peer review only - http://bmjopen.bmj.com/site/about/guidelines.xhtml

For peer review only

Abstract

Objectives

As middle-income countries strive to achieve the Sustainable Development Goals (SDGs), it remains unclear to what degree expanding primary care coverage can help achieve those goals and reduce within-country inequalities in mortality. Our objective was to estimate the potential impact of primary care expansion on cause-specific mortality in the 15 largest Brazilian cities.

Design

Microsimulation model

Setting

15 largest cities by population size in Brazil

Participants

Simulated populations

Interventions

We performed survival analysis to estimate hazard ratios of death by cause and by demographic group, from a national administrative database linked to the Estratégia de Saúde da Família (Family Health Strategy, FHS) electronic health and death records among 1.2 million residents of Rio de Janeiro (2010-2016). We incorporated the hazard ratios into a microsimulation to estimate the impact of changing primary care coverage in the 15 largest cities by population size in Brazil.

Primary and secondary outcome measures

Crude and age-standardized mortality by cause, infant mortality, and under-5 mortality.

Results

Increased FHS coverage would be expected to reduce inequalities in mortality among cities (from 2.8 to 2.4 deaths per 1,000 between the highest- and lowest-mortality city, given a 40-percentage point increase in coverage), between welfare recipients and non-recipients (from 1.3 to 1.0 deaths per 1,000), and among race/ethnic groups (between Black and White Brazilians from 1.0 to 0.8 deaths per 1,000). Even a 40-percentage point increase in coverage, however, would be insufficient to reach SDG targets alone, as it would be expected to reduce premature

For peer review only - http://bmjopen.bmj.com/site/about/guidelines.xhtml
For peer review only

mortality from non-communicable diseases by 20% (versus the target of 33%), and communicable diseases by 15% (versus 100%).

Conclusions

FHS primary care coverage may be critically beneficial to reducing within-country health inequalities, but reaching SDG targets will likely require coordination between primary care and other sectors.

For peer review only

For peer review only - http://bmjopen.bmj.com/site/about/guidelines.xhtml

Strengths and limitations of this study

This study quantified the degree to which expansion or contraction of Brazil's largest primary care program would be expected to help achieve the SDGs, and the implications of program expansion or contraction on inequalities.

The findings suggest that primary care coverage may be critically beneficial to reducing within-country health inequalities, but reaching the Sustainable Development Goal (SDG) targets would be unlikely without additional resources and efforts from other sectors.

The study helps direct emphasis towards coordination between primary care and other sectors, including efforts to address the wider socioeconomic determinants of health.

The principal limitations arise from being based on a simulation model that cannot account for unobserved confounders.

Additionally, the infant mortality and under-5 mortality outcomes were based on hazard ratios from the literature rather than from detailed individual-level data, due to limitations in data availability from the primary datasets we used.

For peer review only

For peer review only - http://bmjopen.bmj.com/site/about/guidelines.xhtml

Introduction

Increasing access to primary care has been linked to reduced mortality at both the individual and population levels.[1–7] Primary care expansion in low- and middle-income countries remains a major strategy for reducing mortality, and for achieving the United Nations Sustainable Development Goals (SDGs).[8] Indeed, primary care expansion is listed by the United Nations as a key intervention to achieve the SDG mortality reduction targets, which include reducing premature mortality from non-communicable diseases by one-third, ending deaths from communicable diseases, and reducing under-5 mortality to <25 per 1,000 live births by the year 2030.[9]

The degree to which improving primary care access can further contribute to reductions in mortality remains unclear. Better evidence is needed to drive policy-making, given that global mortality targets are often out of sync with local health system planning and budgeting activities and insufficiently tailored to local contexts.[10,11] Divestments by national governments from the primary care sector have partially been justified by the lack of clear evidence that primary care can be expected to achieve targets such as the SDGs.[12–14] Local governments often vary in their ability and willingness to fund primary care independently of federal support. Hence, particularly in countries with decentralized decision-making, it is vital to estimate expected declines in mortality from primary care investments, and conversely whether targets can be better designed with local and regional baseline conditions and primary care effectiveness in mind.[15]

In this study, we estimate the potential impact of primary care expansion on mortality by cause of death and by age in the 15 largest Brazilian cities. We use a simulation model incorporating micro-level demographic, health, and effect-size data from Brazil's Estratégia de Saúde da Família (Family Health Strategy, FHS) primary care program. The FHS is Brazil's main strategy for achieving universal health care, and is based on primary care delivery through multidisciplinary care teams co-located in clinics; mobile community healthcare worker teams trained to extend clinic reach; and evidence-based training, protocol management, and record-keeping systems including a unique administrative dataset for tracking health outcomes among individual FHS users and non-users.[1,2] Each team includes a physician, nurses and community healthcare workers responsible for delivering maternal and child healthcare, curative care, health

For peer review only - http://bmjopen.bmj.com/site/about/guidelines.xhtml

promotion and prevention, chronic disease management, home visits, and referrals to a catchment population of approximately 1,000 families (~3,450 individuals). In 2020, 5,462 local government municipalities (out of 5,565 in Brazil) had these health teams, covering 133.9 million individuals (63.7% of the population).[16] Since 1996, municipal governments have been responsible for financing and delivery of primary care in Brazil. In the context of national government cuts to healthcare budgets, local governments have varied in the degree to which they augment primary care investments.[12,13]

Our prior work has shown that expanded FHS coverage in Brazil has been associated with reductions in both non-communicable and communicable disease mortality, infant and under-5 mortality, as well as health disparities among race/ethnic and urban/rural groups.[1,2,7,12] Here, we simulate the fifteen largest Brazilian cities with detailed demographic and health data--incorporating information on variations in FHS primary care coverage, and estimate relationships among coverage to mortality risk at an individual level by cause to project how further expansion or contraction of the FHS program may affect crude and age-standardized mortality by cause, infant mortality, and under-5 mortality, and to compare such mortality rate variations to international targets for mortality. We focus on cities because they have an average FHS coverage of 35% -- far below the national coverage --yet constitute the largest proportion of under-served favela (slum) populations who are the intended primary target populations for FHS.[1]

For peer review only

For peer review only - http://bmjopen.bmj.com/site/about/guidelines.xhtml

Methods

Model structure

We designed and implemented a microsimulation model,[17] which simulates individual people in each of the largest fifteen Brazilian cities, their demographics, their risk of specific causes of death conditional on their location and demographics, and the estimated change in their risk of death given expansion or contraction of the FHS primary care program, over the period 2020-2030. A microsimulation is a model that can be envisioned as a large table, where each row is an individual and each column is a characteristic (i.e., location, demographic, health status) of the individual. Within the microsimulation, probabilities of death by cause by year (derived from annual mortality rate by cause) are conditioned on the location and demographics of the individual, as well as whether they have access to the FHS primary care program. We adjusted those probabilities of death to simulate changes in FHS coverage, per the data sources detailed below.

Relationship between FHS primary care coverage and mortality outcomes

FHS effects estimates were obtained from a previous retrospective study on the association between FHS usage and mortality by age, socioeconomic status, and cause of death for 1.2 million residents of Rio de Janeiro (2010-2016).[18] Specifically, a survival analysis was carried out using the linked *Cadastro Único* national administrative database,[19] FHS electronic health records and mortality records. Flexible parametric survival analysis models were used to estimate hazard ratios for each ICD-10-CM cause of death and cause of death disease group (e.g., Neoplasms are ICD-10 codes C00 through D48[20]) by sex, race, and if the family receives benefits from the national conditional cash transfers program Bolsa Familia or not. The models were inverse probability treatment weighted to adjust for the probability of FHS participation, and regression adjusted for age, highest level of education, disability, unemployment, household per capita income decile, number of family members per bedroom, family size, number of children in family, household flooring, household piped water access, quintiles of household expenditure on medicines, quintile of per capita household expenditure on food, formal labour employment, and if the individual has been hospitalised before FHS use. Results are displayed in

For peer review only - http://bmjopen.bmj.com/site/about/guidelines.xhtml

Supporting Information Figure 1. By contrast with cause-specific adult mortality, hazard ratios for infant mortality and under-5 mortality were obtained from a prior systematic review of literature, as routine administrative data were not available in disaggregated form for children.[21]

Simulation of changing FHS primary care coverage levels

We used data from the Brazilian Institute of Geography and Statistics (IBGE) on fifteen Brazilian cities (including Rio de Janeiro) to generate a simulated population of each city.[22] We generated the simulated representative population based on the demographic characteristics, FHS enrollment probability and mortality risks of each population, as itemized in **Table 1**. We then varied the FHS coverage level in each city, and applied the hazard ratios estimated from the Rio de Janeiro survival analysis (described above) to estimate the impact of changing FHS coverage on mortality in all of the simulated cities, through the method specified below.

We specifically estimated the impact of changing coverage on mortality in two steps. First, we calculated the base mortality probability for each simulated person in the absence of FHS primary care, using the following formula:

[Eq. 1] $m_b \times HR \times p + m_b \times (1 - p) = m_c$,

where m_b is the base mortality probability for each cause of death in each city without FHS (to be estimated); *HR* is the hazard ratio for that cause of death for FHS users versus non-users obtained from the Rio de Janeiro survival analysis; *p* is the latest (2016 observed) FHS-covered proportion of the population in the simulated city, and m_c is the latest (2016 observed) mortality probability in that city. We used 2016 because it was the last year of data available for the Rio de Janeiro analysis.[18]

Second, after calculating the base mortality probability for each cause of death in each city, we then estimated the new mortality probability from equation 1, conditional on a simulated FHS coverage level, to re-estimate the deaths by cause under different FHS coverage levels. We varied the FHS coverage level from 20 percentage points below the current observed coverage level to 40 percentage points above the current observed coverage level in each city (up to a maximum of 100 percent). The choice of 40 percentage points assumed that the FHS expansion policy would have been coordinated at the federal level, with an effort to progress from 60% to 100% coverage by 2030.

For peer review only - http://bmjopen.bmj.com/site/about/guidelines.xhtml

The primary outcome was change in all-cause mortality. Secondary outcomes included changes in cause-specific and all-cause mortality subgrouped by race/ethnicity, and whether the family receives Bolsa Familia benefits or not, considering the differential hazard ratios of death by cause and by these characteristics, as described above.

Uncertainty analyses

At each level of simulated FHS coverage, we repeatedly simulated each of the fifteen cities' populations a total of 10,000 times each. During each of these simulations, we sampled with replacement from random normal distributions constructed around the mean and 95% confidence intervals around the mean of the hazard ratios of FHS primary care enrollment on each cause of death (**Supporting Materials Figure 1, Supporting Materials Table 1**), to generate uncertainty intervals around our outcome estimates. All analyses were performed in *R*.

Funding statement

This work was supported by a grant from the Health Systems Research Initiative with funding from the Foreign, Commonwealth and Development Office, the UK Medical Research Council and Wellcome, in collaboration with the UK Economic and Social Research Council (grant no. MR/P014593/1). The funder had no role in study design; collection, analysis, and interpretation of data; report writing; or decision to submit the paper for publication.

Patient and public involvement

No patient involved.

For peer review only

For peer review only - http://bmjopen.bmj.com/site/about/guidelines.xhtml

Results

Population characteristics

Population demographics, FHS primary care program coverage rates, and mortality rate estimates by city are provided in **Table 1**. Notable demographics included the population aged less than 15 years (which varied between 18.8% to 29.9%), the populations aged above age 64 years (between 3.2% and 7.7%), race/ethnicity (between 14.8% and 78.0% identifying as White, between 2.3% and 38.0% Black, and 10.2% to 77.0% Pardo). City poverty rates varied from 1.7% to 15.6%. Age-standardized all-cause mortality rates varied from 4.9 to 7.0 per 1,000 population, infant mortality varied from 11.8 to 22.0 per 1,000 live births, and under-5 mortality from 12.0 to 24.0 per 1,000 live births. (Hence, all cities had under-5 mortality rates below 25 per 1,000 live births, meeting SDG target 3.2).

Projected mortality variations with changes in primary care coverage

In our simulations, we varied the FHS coverage level from 20 percentage points below the current observed coverage level to 40 percentage points above the current observed coverage level in each city (up to a maximum of 100 percent). Projected variations in crude and age-standardized all-cause mortality, as well as in infant mortality and under-5 mortality, are illustrated in **Figures 1 and 2**. Variations in cause-specific age-standardized mortality are itemized in **Table 2**. Uncertainty estimates (95% confidence intervals) around all estimates are provided in **Supporting Information Figure 2** and **Supporting Information Table 2**. In general, increases in FHS coverage were associated with reductions in predicted mortality.

As shown in **Figure 1A and 1B**, increases in FHS coverage would be expected to contribute to reductions in inequalities between cities in both crude- and all-cause mortality. The associations between FHS coverage and reductions in mortality were greatest in cities with the highest rates of baseline mortality. For example, a 20-percentage point decline in coverage would be expected to increase crude mortality in Rio de Janeiro by 12% (from a current level of 5.9 to 6.6 per 1,000 [95% CI: 6.2, 7.0]), and increase crude mortality in Sao Paulo by 10% (from 4.8 to 5.3 per 1,000 [95% CI: 5.2, 5.4]). By contrast, as shown in **Figure 1**, when primary care coverage rates increased, the model observed less inequality among cities, with diminishing

For peer review only - http://bmjopen.bmj.com/site/about/guidelines.xhtml

returns on further reductions in mortality among those already with low mortality rates. For example, a 20-percentage point increase in coverage would be expected to reduce crude mortality by 21% in Sao Paulo (from 4.8 to 3.8 per 1,000 [95% CI: 3.3, 4.4]) and by 24% in Rio de Janeiro (from 5.9 to 4.5 per 1,000 [95% CI: 3.4, 5.5]). A 40-percentage point increase in coverage would be expected to reduce overall between-city all-cause mortality (differences in mortality between the highest and lowest mortality city) from 2.8 to 2.4 deaths per 1,000 (95% CI: 2.3, 2.6). The same pattern was observed with infant mortality (**Figure 1C**) and under-5 mortality (**Figure 1D**).

As shown in **Table 2**, the specific causes of mortality that were most sensitive to the changes in FHS primary care coverage were chronic non-communicable disease deaths, including cardiovascular disease deaths and deaths from injury (n.b., many FHS clinics are often the first point of service for injuries). According to the model, a 20-percentage point reduction in coverage would be expected to raise deaths from unintentional and intentional injuries by 13% (95% CI: 9%, 17%) and 16% (95% CI: 13%, 20%), respectively, and from heart disease and stroke from 11% (95% CI: 7%, 14%) and 14% (95% CI: 10%, 17%), respectively. Conversely, the smallest changes in cause-specific mortality were for deaths from nervous system diseases, tuberculosis, malaria, neglected tropical diseases, and maternity. For the SDG of reducing by one-third premature mortality (death prior to age 70 years) from non-communicable diseases, the results implied that even an increase in FHS primary care coverage by 40 percentage points would still insufficient by itself to reach the target, as premature mortality from non-communicable diseases was only estimated to fall by 20% (95% CI: 7%, 34%). For the SDG of ending deaths from communicable diseases, the model results implied that an increase in FHS primary care coverage by 40 percentage points would be expected to reduce mortality from communicable diseases by 15% (95% CI: 1%, 29%).

Subgroup analyses

The model was used to estimate changes in mortality across groups defined by race/ethnicity and whether the family receives Bolsa Familia benefits, considering the differential hazard ratios of death by cause and by these characteristics (**Supporting Information Table 1**). Across all causes of death, FHS primary care coverage disproportionately

For peer review only - http://bmjopen.bmj.com/site/about/guidelines.xhtml

benefited Black and Pardo groups, and those on Bolsa Familia benefits. For each percentage point decline (or increase) in primary care FHS coverage, the absolute increase (or decrease) in mortality was 1.3 times higher for Bolsa Familia families than for non-recipient families, 1.2 times higher for Black and 1.1 times higher for Pardo families than for White families (**Figure 2**). A 40-percentage point increase in coverage would be expected to reduce inequality in mortality between welfare recipients and non-recipients from 1.3 to 1.0 deaths per 1,000 (95% CI: 0.8, 1.2), inequality between Black and White Brazilians from 1.0 to 0.8 deaths per 1,000 (95% CI: 0.6, 0.9), and inequality between Pardo and White Brazilians from 0.3 to 0.2 per 1,000 (95% CI: 0.1, 0.3).

For peer review only

For peer review only - http://bmjopen.bmj.com/site/about/guidelines.xhtml

Discussion

We simulated the impacts of changes in FHS coverage on mortality in the fifteen largest Brazilian cities using detailed demographic and health data, and estimated relationships at an individual level by cause of death, controlling for major covariates. We found reductions in FHS coverage would be expected to lead to higher mortality and exacerbate inequalities among cities. Additionally, marginalized groups including those receiving Bolsa Familia and those of minority race/ethnic groups would be expected to disproportionately benefit from increasing FHS coverage, and so increased FHS coverage would also be expected to drive reductions in inequalities within cities. We estimated that an increase in FHS primary care coverage by 40 percentage points would still be insufficient on its own, however, to reach SDG target 3.4, as it would only reduce premature mortality from non-communicable diseases by 20% (versus the SDG target of 33%). Additionally, our results implied that even an increase in FHS primary care coverage by 40 percentage points would be expected to reduce mortality from communicable diseases by 15% but still be far from the SDG of ending deaths from communicable diseases. By contrast, Brazilian cities had already reached SDG target 3.2 of reducing under-5 mortality to less than 25 per 1,000 live births.[23]

The study findings suggest that, in the context of a health system with decentralized decision-making to over 5,000 municipal governments, geographic inequality in mortality in Brazil will potentially be greatly affected by future federal financing and support of the FHS. Investment in primary care may be beneficial for local achievement of SDGs for both non-communicable and communicable diseases and reduce geographic inequality in these outcomes. Nonetheless, localities would need to engage with non-health sectors to achieve an elimination of health inequalities and should consider setting bolder local targets for accelerating under-5 mortality rates beyond those prescribed in the SDGs.

Our results are particularly important considering the current economic crisis due to the COVID-19 pandemic in Brazil, which is already responsible for a dramatic increase in poverty and unemployment and will have long-term effects on the most vulnerable groups of the population.[24] Our findings are consistent with previous studies which have shown a synergistic impact of FHS with Bolsa Familia (the country's social welfare program) on child mortality,[25,26] and on premature mortality during the past Brazilian economic recession.[27] Other simulation

For peer review only - http://bmjopen.bmj.com/site/about/guidelines.xhtml

studies, performed at the aggregate-level, have also indicated how a combined expansion of FHS and Bolsa Familia during periods of economic crisis are able to reduce the number of childhood deaths.[12] While our study is focused on Brazilian cities, recent literature has also demonstrated the effectiveness of interventions to expand the primary care coverage to rural areas, as the Mais Medicos Program, on the reduction of premature mortality,[28] and have shown how weakening of such intervention during periods of economic austerity could be responsible for a large number of avoidable deaths.[14,29]

Brazil is one of the few low and middle-income countries with a universal healthcare system, the Unified Health System (SUS), based on one of the world's largest primary health care programs, the FHS. The expansion of the SUS and FHS during the last 30 years was responsible for large reductions on mortality and health inequalities,[30] but is currently under threat by aggressive and long-term fiscal austerity measures that could undermine its consolidation and even reduce its dimension and effectiveness,[13,14] so robust evidence on the impact of its components -- including FHS, are urgently needed.

Our study has several strengths and limitations. Our simulation model used estimates of FHS impact on adult mortality based on a unique, individual-level dataset with a rich set of covariates including key socioeconomic and health variables that relate to mortality; nevertheless, factors other than those we have controlled for may be additionally important to consider and may serve as unmeasured factors influencing our results, such as prior family history of disease. Additionally, this dataset was restricted to the city of Rio de Janeiro where FHS impacts may differ from those in the other major cities included in our study. Indeed, the FHS in Rio has major investments in clinics and equipment, a residency programme, and higher salaries for doctors. Our simulation of infant mortality and under-5 mortality was based on hazard ratios from the literature rather than from detailed individual-level data, due to limitations in data availability from the primary datasets we used. This increases the possibility that inequalities across key socioeconomic groups would be underestimated by using a group measure, and hence the overall impact of the primary care program may be underestimated.

In future research, we plan to examine how rural populations respond differently to urban populations to FHS primary care coverage expansion and identify factors that may help enhance the effectiveness of FHS in reducing mortality across Brazil. Our work also suggests future

For peer review only

For peer review only - http://bmjopen.bmj.com/site/about/guidelines.xhtml

research should include more comparative analysis of the health impacts delivered by different models of primary care in Latin America and worldwide. In the meantime, our results suggest that increasing primary care coverage may be critically beneficial to achieving SDG targets including reducing within-country inequalities between geographical areas, income and race/ethnic groups in Brazil.

For peer review only

For peer review only - http://bmjopen.bmj.com/site/about/guidelines.xhtml

Competing interests

None.

Author contributions

CM and DR conceived of the study. VS, AT, and BD collected data. SB, TVH, and DR performed the analysis. SB wrote the draft. All authors contributed to editing of the manuscript.

Data sharing

Technical appendix, statistical code, and dataset available from https://github.com/sanjaybasu concurrent with publication.

Ethics Statement

Approval for this study was obtained from the Brazilian National Commission for Ethics in Research (Comissão Nacional de Ética em Pesquisa [CONEP]; number 2.689.528) and Imperial College London's Ethical Committee.

For peer review only

For peer review only - http://bmjopen.bmj.com/site/about/guidelines.xhtml

References

1 Hone T, Rasella D, Barreto M, Atun R, Majeed A, Millett C. Large Reductions In Amenable Mortality Associated With Brazil's Primary Care Expansion And Strong Health Governance. Health Affairs. 2017; **36**: 149–58.

2 Hone T, Rasella D, Barreto ML, Majeed A, Millett C. Association between expansion of primary healthcare and racial inequalities in mortality amenable to primary care in Brazil: A national longitudinal analysis. PLOS Medicine. 2017; **14**: e1002306.

3 Shi L, Macinko J, Starfield B, Wulu J, Regan J, Politzer R. The relationship between primary care, income inequality, and mortality in US States, 1980-1995. *J Am Board Fam Pract* 2003; **16**: 412–22.

4 Shi L, Macinko J, Starfield B, Politzer R, Xu J. Primary care, race, and mortality in US states. Social Science & Medicine. 2005; **61**: 65–75.

5 Institute of Medicine, Board on Population Health and Public Health Practice, Committee on Integrating Primary Care and Public Health. Primary Care and Public Health: Exploring Integration to Improve Population Health. National Academies Press, 2012 https://books.google.com/books/about/Primary_Care_and_Public_Health.html?hl=&id=qt1dP5ia6vwC.

6 Basu S, Berkowitz SA, Phillips RL, Bitton A, Landon BE, Phillips RS. Association of Primary Care Physician Supply With Population Mortality in the United States, 2005-2015. *JAMA Intern Med* 2019; **179**: 506–14.

7 Rasella D, Harhay MO, Pamponet ML, Aquino R, Barreto ML. Impact of primary health care on mortality from heart and cerebrovascular diseases in Brazil: a nationwide analysis of longitudinal data. *BMJ* 2014; **349**: g4014.

8 Hone T, Macinko J, Millett C. Revisiting Alma-Ata: what is the role of primary health care in achieving the Sustainable Development Goals? The Lancet. 2018; **392**: 1461–72.

9 Pettigrew LM, De Maeseneer J, Anderson M-IP, Essuman A, Kidd MR, Haines A. Primary health care and the Sustainable Development Goals. The Lancet. 2015; **386**: 2119–21.

10 Nilsson M, Griggs D, Visbeck M. Policy: Map the interactions between Sustainable Development Goals. 2016; published online June 15. DOI:10.1038/534320a.

11 Siddiqi S, Aftab W, Siddiqui FJ, *et al.* Global strategies and local implementation of health and health-related SDGs: lessons from consultation in countries across five regions. *BMJ Global Health* 2020; **5**: e002859.

12 Rasella D, Basu S, Hone T, Paes-Sousa R, Ocké-Reis CO, Millett C. Child morbidity and mortality associated with alternative policy responses to the economic crisis in Brazil: A

For peer review only - http://bmjopen.bmj.com/site/about/guidelines.xhtml

nationwide microsimulation study. PLOS Medicine. 2018; **15**: e1002570.

13 Souza LEPF de, de Souza LEPF, de Barros RD, *et al.* The potential impact of austerity on attainment of the Sustainable Development Goals in Brazil. BMJ Global Health. 2019; **4**: e001661.

14 Rasella D, Hone T, de Souza LE, Tasca R, Basu S, Millett C. Mortality associated with alternative primary healthcare policies: a nationwide microsimulation modelling study in Brazil. *BMC Med* 2019; **17**: 82.

15 Dalby S, Horton S, Mahon R. Global governance challenges in achieving the Sustainable Development Goals. Achieving the Sustainable Development Goals. 2019; : 1–18.

16 Informacio e Gestao da Atencao Basica. Cobertua da Atencao Basica. https://egestorab.saude.gov.br/paginas/acessoPublico/relatorios/relHistoricoCoberturaAB.xhtml (accessed Nov 28, 2020).

17 Basu S. Microsimulation. Oxford Medicine Online. 2017. DOI:10.1093/med/9780190667924.003.0008.

18 Hone T, Saraceni V, Medina Coeli C, *et al.* Primary healthcare expansion and mortality in Brazil's urban poor: A cohort analysis of 1.2 million adults. *PLoS Med* 2020; **17**: e1003357.

19 Brazil's Cadastro Unico: an enduring bridge to consolidated targeting efforts. https://socialprotection.org/discover/publications/brazil%E2%80%99s-cadastro-unico-enduring-bridge-consolidated-targeting-efforts (accessed Sept 30, 2020).

20 WHO | International Classification of Diseases (ICD) Information Sheet. 2019; published online Oct 11. http://www.who.int/classifications/icd/factsheet/en/ (accessed Sept 30, 2020).

21 Bastos ML, Menzies D, Hone T, Dehghani K, Trajman A. The impact of the Brazilian family health on selected primary care sensitive conditions: A systematic review. *PLoS One* 2017; **12**: e0182336.

22 IBGE. https://www.ibge.gov.br/ (accessed Nov 29, 2020).

23 Datasus. DATASUS. http://www2.datasus.gov.br/DATASUS/index.php?area=0205 (accessed Nov 29, 2020).

24 Marson FAL, Ortega MM. COVID-19 in Brazil. *Pulmonology* 2020; **26**: 241–4.

25 Guanais FC. The Combined Effects of the Expansion of Primary Health Care and Conditional Cash Transfers on Infant Mortality in Brazil, 1998–2010. *Am J Public Health* 2015; **105**: S593–9.

26 Rasella D, Aquino R, Santos CAT, Paes-Sousa R, Barreto ML. Effect of a conditional cash

For peer review only

For peer review only - http://bmjopen.bmj.com/site/about/guidelines.xhtml

transfer programme on childhood mortality: a nationwide analysis of Brazilian municipalities. *Lancet* 2013; **382**: 57–64.

27 Hone T, Mirelman AJ, Rasella D, *et al.* Effect of economic recession and impact of health and social protection expenditures on adult mortality: a longitudinal analysis of 5565 Brazilian municipalities. *Lancet Glob Health* 2019; **7**: e1575–83.

28 Hone T, Powell-Jackson T, Santos LMP, *et al.* Impact of the Programa Mais médicos (more doctors Programme) on primary care doctor supply and amenable mortality: quasi-experimental study of 5565 Brazilian municipalities. *BMC Health Serv Res* 2020; **20**: 873.

29 Francesconi GV, Tasca R, Basu S, Rocha TAH, Rasella D. Mortality associated with alternative policy options for primary care and the Mais Médicos (More Doctors) Program in Brazil: forecasting future scenarios. *Rev Panam Salud Publica* 2020; **44**: e31.

30 Castro MC, Massuda A, Almeida G, *et al.* Brazil's unified health system: the first 30 years and prospects for the future. *Lancet* 2019; **394**: 345–56.

For peer review only

For peer review only - http://bmjopen.bmj.com/site/about/guidelines.xhtml

Declaration of Interests

None

Acknowledgements

This work was supported by a grant from the Health Systems Research Initiative with funding from the Foreign, Commonwealth and Development Office, the UK Medical Research Council and Wellcome, in collaboration with the UK Economic and Social Research Council (grant no. MR/P014593/1).

For peer review only

For peer review only - http://bmjopen.bmj.com/site/about/guidelines.xhtml

Tables and Figures

Table 1: Demographics, primary care coverage, and mortality among the fifteen largest Brazilian cities. FHS: Estratégia de Saúde da Família (Family Health Strategy) primary care program (2016).[19]

City	Age, less than 15 years, %	Age, greater than 64 years, %	Female, %	Race, % White	Race, % Black	Ethnicity, % Pardo	Income, % below poverty line	Education, % graduated secondary school (age 18-20)	FHS primary care coverage, %	Mortality, all cause, crude (per 1,000)	Mortality, all cause, age adjusted (per 1,000)	Infant mortality (per 1,000 live births)	Under-5 mortality (per 1,000 live births)
Belo Horizonte	29.9	4.7	52.7	40.5	12.6	46.5	5.6	47.5	76.5	5.76	4.95	13.0	15.2
Belém	24.8	5.5	52.1	21.2	9.1	69.3	14.9	37.1	23.5	5.49	6.17	16.1	17.2
Brasília	23.7	5.0	52.2	40.0	10.6	48.3	4.9	53.5	37.8	3.93	4.98	14.0	15.8
Campinas	19.3	5.8	51.8	57.2	8.9	30.8	3.2	53.2	44.5	5.60	4.86	11.8	13.7
Curitiba	20.0	4.9	52.3	74.3	3.8	58.5	1.7	57.8	36.4	5.19	4.86	11.9	13.6
Fortaleza	22.6	4.9	53.2	32.3	5.8	61.4	12.1	45.4	45.9	4.93	5.40	15.8	16.9
Goiânia	20.8	4.2	52.3	43.2	6.4	49.8	3.1	57.0	44.3	5.42	5.99	13.1	15.0
Maceió	25.0	4.4	53.2	27.1	5.6	66.7	15.6	42.6	29.0	5.91	7.04	22.0	24.0
Manaus	28.2	3.2	51.2	20.1	2.3	77.0	12.9	38.8	27.0	4.34	6.84	14.2	15.2
Porto Alegre	18.8	6.4	53.6	78.0	11.4	10.2	3.8	48.2	55.0	7.42	5.43	11.6	13.1
Recife	20.9	5.5	53.8	36.9	9.1	52.7	13.2	46.7	54.8	6.43	6.05	15.6	12.5
Rio de Janeiro	19.4	7.6	53.2	48.5	11.6	39.2	5.0	45.9	62.9	8.14	5.97	13.0	14.6
Salvador	20.7	4.3	53.3	14.8	38.0	46.8	11.4	41.8	26.7	5.32	5.90	14.9	12.0
São Luís	23.7	3.8	53.2	19.9	15.5	63.9	13.8	53.1	34.4	4.64	5.91	18.1	19.8
São Paulo	20.8	5.5	52.6	57.9	8.7	30.4	4.3	50.5	35.4	5.71	5.09	13.2	14.7

For peer review only - http://bmjopen.bmj.com/site/about/guidelines.xhtml

Table 2: Relative impact on cause-specific mortality given changes in the percentage point coverage in the FHS primary care strategy (Estratégia de Saúde da Família). The cells show the ratio of mortality by cause under different levels of FHS coverage, compared to the current mortality rate (at 0% change in FHS coverage), the reference column. 95% confidence intervals in Supporting Information Table 2.

	**Ratio of mortality by cause, compared to current mortality rate (at 0%)**						
**Percentage point change in FHS coverage:**	**-20%**	**-10%**	**0%**	**10%**	**20%**	**30%**	**40%**
All causes	1.11	1.05	1.00	0.95	0.89	0.84	0.78
Infections (excluding HIV, TB, malaria, NTDs)	1.11	1.05	1.00	0.95	0.89	0.84	0.79
HIV	1.08	1.04	1.00	0.96	0.92	0.88	0.84
TB, malaria, NTDs	1.03	1.02	1.00	0.98	0.97	0.95	0.94
Respiratory	1.10	1.05	1.00	0.95	0.90	0.85	0.80
Nutrition	1.08	1.04	1.00	0.96	0.92	0.87	0.83
Neoplasms	1.11	1.05	1.00	0.95	0.89	0.84	0.78
Nervous system	1.06	1.03	1.00	0.97	0.94	0.92	0.89
Endocrine	1.11	1.05	1.00	0.95	0.89	0.84	0.79
Mental/substance use	1.10	1.05	1.00	0.95	0.90	0.84	0.79
Stroke	1.14	1.07	1.00	0.93	0.86	0.80	0.73
Heart disease	1.11	1.05	1.00	0.95	0.89	0.84	0.79
Other cardiovascular	1.13	1.06	1.00	0.94	0.87	0.81	0.74
Digestive	1.10	1.05	1.00	0.95	0.90	0.84	0.79
Genitourinary	1.10	1.05	1.00	0.95	0.90	0.86	0.81
Unintentional injuries	1.13	1.07	1.00	0.93	0.87	0.80	0.74
Intentional injuries	1.16	1.08	1.00	0.92	0.84	0.75	0.67
Maternal	1.01	1.00	1.00	1.00	0.99	0.99	0.99

For peer review only

For peer review only - http://bmjopen.bmj.com/site/about/guidelines.xhtml

Figure Legends

Figure 1: Projected variations in (A) all-cause crude mortality, (B) all-cause age-standardized mortality, (C) infant mortality and (D) under-5 mortality given different levels of Family Health Strategy (FHS) program primary care coverage. See Table 1 for current coverage levels corresponding to a 0% change on the x-axis. See 95% confidence intervals in Supporting Information Figure 2.

Figure 2: Projected changes in all-cause and cause-specific age-standardized mortality given (A) a 20 percentage point decline or (B) a 40 percentage point increase in Family Health Strategy (FHS) program primary care coverage from the baseline levels indicated in Table 1. Legend: BF: participation in the Bolsa Familia program.

For peer review only

For peer review only - http://bmjopen.bmj.com/site/about/guidelines.xhtml

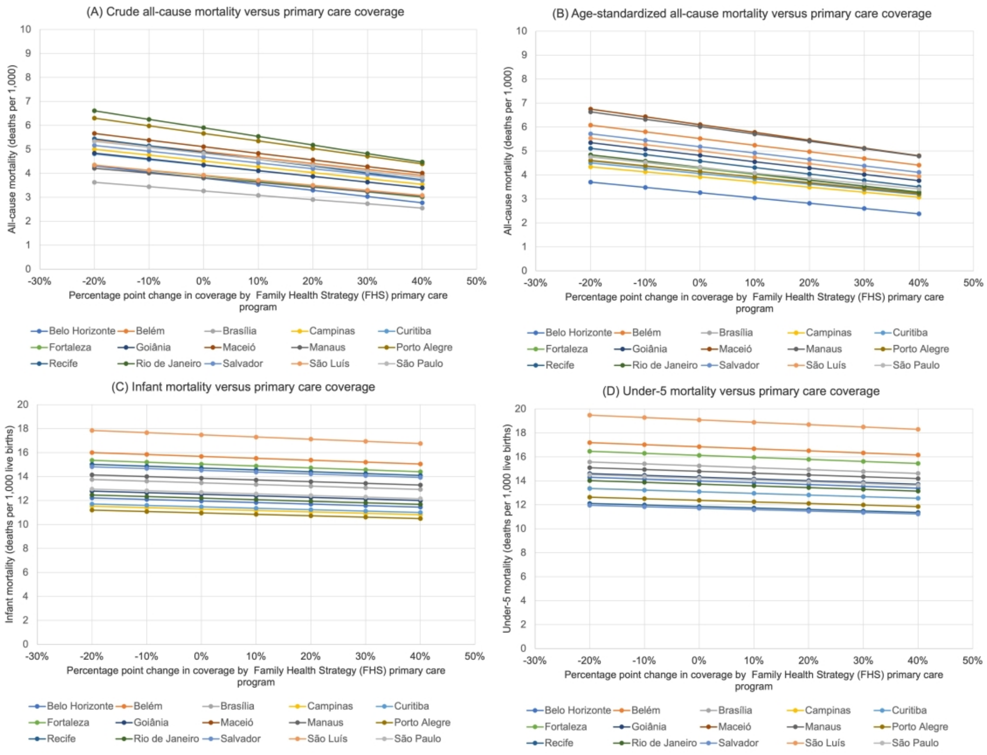

FIGURE 1

417x316mm (330 x 330 DPI)

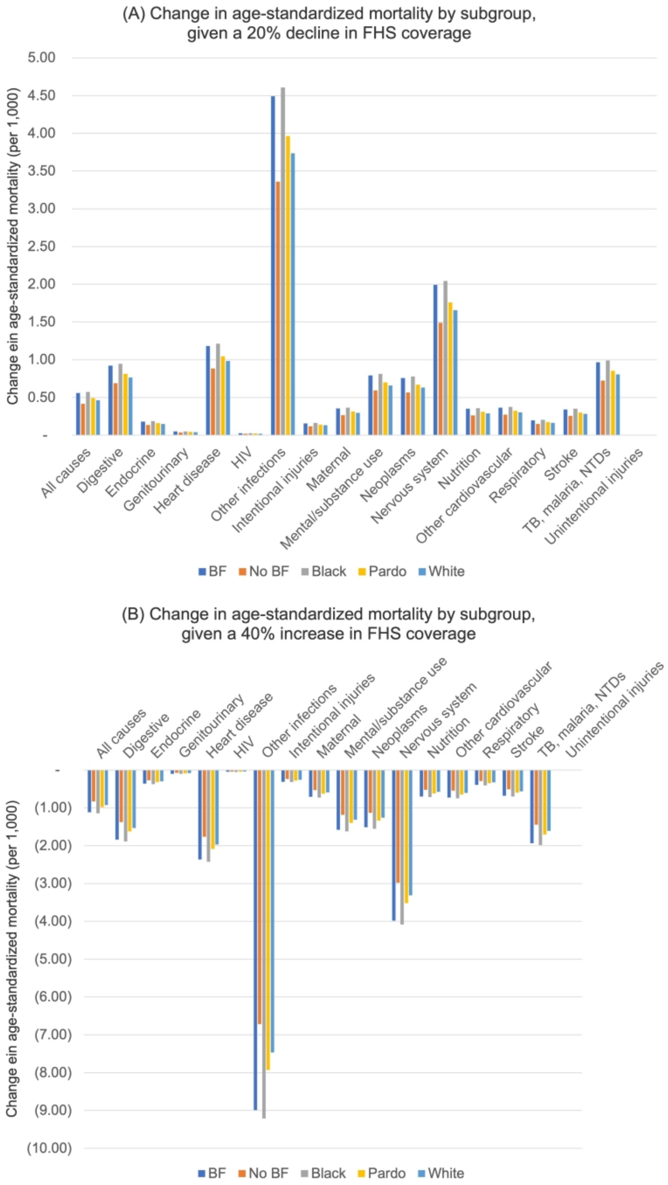

FIGURE 2

203x357mm (330 x 330 DPI)

Supporting Information

For peer review only

For peer review only - http://bmjopen.bmj.com/site/about/guidelines.xhtml

Supporting Information Table 1: Hazard ratios for death by cause group, subdivided by sex, race/ethnicity, and whether or not the family receives Bolsa Familia (BF) benefits.

Cause	Subgroup	Hazard Ratio	Lower 95% Confidence interval	Upper 95% Confidence interval
All Causes	Males	0.68	0.64	0.71
All Causes	Females	0.49	0.47	0.52
All Causes	Race White	0.60	0.56	0.64
All Causes	Race Black	0.50	0.47	0.54
All Causes	Race Pardo	0.57	0.54	0.60
All Causes	No Bf	0.64	0.61	0.67
All Causes	Bf	0.52	0.49	0.54
Infectious And Parasitic Diseases	Males	0.82	0.62	1.08
Infectious And Parasitic Diseases	Females	0.53	0.41	0.67
Infectious And Parasitic Diseases	Race White	0.86	0.63	1.17
Infectious And Parasitic Diseases	Race Black	0.68	0.45	1.02
Infectious And Parasitic Diseases	Race Pardo	0.52	0.39	0.69
Infectious And Parasitic Diseases	No Bf	0.68	0.52	0.88
Infectious And Parasitic Diseases	Bf	0.63	0.49	0.82
Hiv/Aids	Males	1.15	0.88	1.52
Hiv/Aids	Females	0.68	0.55	0.83
Hiv/Aids	Race White	0.67	0.44	1.01
Hiv/Aids	Race Black	0.69	0.51	0.93
Hiv/Aids	Race Pardo	1.02	0.80	1.31
Hiv/Aids	No Bf	1.20	0.86	1.68
Hiv/Aids	Bf	0.73	0.61	0.89

For peer review only - http://bmjopen.bmj.com/site/about/guidelines.xhtml
For peer review only

Tuberculosis, Malaria, And Ntds	Males	0.91	0.61	1.35
Tuberculosis, Malaria, And Ntds	Females	0.24	0.13	0.43
Tuberculosis, Malaria, And Ntds	Race White	0.61	0.29	1.28
Tuberculosis, Malaria, And Ntds	Race Black	0.49	0.25	0.95
Tuberculosis, Malaria, And Ntds	Race Pardo	0.67	0.42	1.06
Tuberculosis, Malaria, And Ntds	No Bf	0.68	0.38	1.22
Tuberculosis, Malaria, And Ntds	Bf	0.56	0.38	0.82
Maternal Causes (Females Only)	Females	0.97	0.60	1.56
Maternal Causes (Females Only)	Race White	1.01	0.46	2.23
Maternal Causes (Females Only)	Race Black	1.09	0.46	2.56
Maternal Causes (Females Only)	Race Pardo	0.82	0.37	1.82
Maternal Causes (Females Only)	No Bf	1.93	0.63	5.90
Maternal Causes (Females Only)	Bf	0.81	0.48	1.39
Nutritional Deficiencies	Males	0.59	0.26	1.33
Nutritional Deficiencies	Females	0.52	0.28	0.98
Nutritional Deficiencies	Race White	0.21	0.05	0.97
Nutritional Deficiencies	Race Black	0.40	0.14	1.19
Nutritional Deficiencies	Race Pardo	0.92	0.50	1.69
Nutritional Deficiencies	No Bf	0.99	0.53	1.88
Nutritional Deficiencies	Bf	0.23	0.10	0.54
Diseases Of The Nervous System	Males	0.52	0.28	0.98
Diseases Of The Nervous System	Females	0.58	0.38	0.91
Diseases Of The Nervous System	Race White	0.46	0.25	0.88

For peer review only - http://bmjopen.bmj.com/site/about/guidelines.xhtml
For peer review only

Diseases Of The Nervous System	Race Black	0.83	0.40	1.75
Diseases Of The Nervous System	Race Pardo	0.55	0.31	0.97
Diseases Of The Nervous System	No Bf	0.48	0.29	0.82
Diseases Of The Nervous System	Bf	0.68	0.41	1.12
Endocrine Disorders	Males	0.74	0.57	0.95
Endocrine Disorders	Females	0.50	0.41	0.61
Endocrine Disorders	Race White	0.58	0.43	0.79
Endocrine Disorders	Race Black	0.55	0.40	0.74
Endocrine Disorders	Race Pardo	0.60	0.47	0.76
Endocrine Disorders	No Bf	0.59	0.47	0.75
Endocrine Disorders	Bf	0.59	0.47	0.73
Mental And Substance Use Disorders	Males	0.66	0.41	1.05
Mental And Substance Use Disorders	Females	0.27	0.15	0.50
Mental And Substance Use Disorders	Race White	0.67	0.32	1.41
Mental And Substance Use Disorders	Race Black	0.17	0.05	0.56
Mental And Substance Use Disorders	Race Pardo	0.53	0.32	0.86
Mental And Substance Use Disorders	No Bf	0.75	0.36	1.54
Mental And Substance Use Disorders	Bf	0.40	0.26	0.61
Stroke	Males	0.77	0.63	0.94
Stroke	Females	0.45	0.38	0.53
Stroke	Race White	0.67	0.53	0.85
Stroke	Race Black	0.45	0.34	0.58
Stroke	Race Pardo	0.60	0.50	0.73

For peer review only

For peer review only - http://bmjopen.bmj.com/site/about/guidelines.xhtml

Stroke	No Bf	0.65	0.54	0.78
Stroke	Bf	0.50	0.42	0.60
Heart Disease	Males	0.59	0.52	0.67
Heart Disease	Females	0.41	0.37	0.46
Heart Disease	Race White	0.47	0.40	0.56
Heart Disease	Race Black	0.43	0.35	0.52
Heart Disease	Race Pardo	0.51	0.44	0.58
Heart Disease	No Bf	0.52	0.46	0.59
Heart Disease	Bf	0.47	0.42	0.54
Other Cardiovascular Diseases	Males	0.68	0.54	0.86
Other Cardiovascular Diseases	Females	0.49	0.41	0.60
Other Cardiovascular Diseases	Race White	0.63	0.47	0.84
Other Cardiovascular Diseases	Race Black	0.41	0.30	0.56
Other Cardiovascular Diseases	Race Pardo	0.65	0.52	0.81
Other Cardiovascular Diseases	No Bf	0.64	0.52	0.79
Other Cardiovascular Diseases	Bf	0.50	0.40	0.62
Digestive Diseases	Males	0.67	0.53	0.84
Digestive Diseases	Females	0.52	0.41	0.66
Digestive Diseases	Race White	0.61	0.46	0.83
Digestive Diseases	Race Black	0.47	0.32	0.70
Digestive Diseases	Race Pardo	0.61	0.48	0.79
Digestive Diseases	No Bf	0.61	0.47	0.79
Digestive Diseases	Bf	0.59	0.48	0.74
Genitourinary Diseases	Males	0.67	0.51	0.88

For peer review only - http://bmjopen.bmj.com/site/about/guidelines.xhtml

Genitourinary Diseases	Females	0.52	0.42	0.65
Genitourinary Diseases	Race White	0.63	0.46	0.87
Genitourinary Diseases	Race Black	0.60	0.43	0.83
Genitourinary Diseases	Race Pardo	0.57	0.44	0.74
Genitourinary Diseases	No Bf	0.64	0.51	0.80
Genitourinary Diseases	Bf	0.52	0.40	0.68
Unintentional Injuries	Males	0.56	0.43	0.74
Unintentional Injuries	Females	0.43	0.32	0.58
Unintentional Injuries	Race White	0.50	0.33	0.76
Unintentional Injuries	Race Black	0.47	0.31	0.71
Unintentional Injuries	Race Pardo	0.53	0.40	0.71
Unintentional Injuries	No Bf	0.55	0.40	0.78
Unintentional Injuries	Bf	0.46	0.36	0.60
Intentional Injuries	Males	0.42	0.33	0.53
Intentional Injuries	Females	0.30	0.18	0.50
Intentional Injuries	Race White	0.51	0.33	0.79
Intentional Injuries	Race Black	0.45	0.27	0.73
Intentional Injuries	Race Pardo	0.34	0.24	0.46
Intentional Injuries	No Bf	0.72	0.51	1.01
Intentional Injuries	Bf	0.28	0.21	0.37
Neoplasms	Males	0.97	0.86	1.11
Neoplasms	Females	0.61	0.55	0.68
Neoplasms	Race White	0.68	0.59	0.78
Neoplasms	Race Black	0.69	0.57	0.84
Neoplasms	Race Pardo	0.83	0.74	0.93

For peer review only - http://bmjopen.bmj.com/site/about/guidelines.xhtml

Neoplasms	No Bf	0.77	0.69	0.86
Neoplasms	Bf	0.73	0.65	0.82
Respiratory Infections And Diseases	Males	0.84	0.73	0.98
Respiratory Infections And Diseases	Females	0.49	0.42	0.56
Respiratory Infections And Diseases	Race White	0.73	0.62	0.87
Respiratory Infections And Diseases	Race Black	0.57	0.44	0.72
Respiratory Infections And Diseases	Race Pardo	0.58	0.49	0.69
Respiratory Infections And Diseases	No Bf	0.68	0.59	0.79
Respiratory Infections And Diseases	Bf	0.61	0.52	0.70

For peer review only

For peer review only - http://bmjopen.bmj.com/site/about/guidelines.xhtml

Supporting Information Table 2: (A) 95% lower confidence interval estimates and (B) 95% upper confidence interval estimates around the relative impact on cause-specific mortality given changes in the percentage point coverage in the FHS program (Estratégia de Saúde da Família) primary care program. The cells show the ratio of mortality by cause under different levels of FHS coverage, compared to the current mortality rate (at 0% change in FHS coverage), the reference column.

For peer review only

For peer review only - http://bmjopen.bmj.com/site/about/guidelines.xhtml

(A)

	Ratio of mortality by cause, compared to current mortality rate (at 0%)						
**Percentage point change in FHS coverage:**	**-20%**	**-10%**	**0%**	**10%**	**20%**	**30%**	**40%**
All causes	1.07	1.00	0.93	0.86	0.79	0.72	0.65
Infections (excluding HIV, TB, malaria, NTDs)	1.07	1.00	0.93	0.86	0.79	0.72	0.66
HIV	1.04	0.99	0.93	0.88	0.82	0.76	0.70
TB, malaria, NTDs	1.00	0.97	0.93	0.90	0.86	0.82	0.78
Respiratory	1.06	1.00	0.93	0.87	0.80	0.73	0.67
Nutrition	1.05	0.99	0.93	0.87	0.81	0.75	0.69
Neoplasms	1.07	1.00	0.93	0.86	0.79	0.72	0.65
Nervous system	1.02	0.98	0.93	0.89	0.84	0.79	0.74
Endocrine	1.07	1.00	0.93	0.86	0.79	0.72	0.65
Mental/substance use	1.07	1.00	0.93	0.86	0.80	0.73	0.66
Stroke	1.10	1.01	0.93	0.85	0.77	0.69	0.61
Heart disease	1.07	1.00	0.93	0.86	0.80	0.73	0.66
Other cardiovascular	1.09	1.01	0.93	0.85	0.77	0.70	0.62
Digestive	1.07	1.00	0.93	0.86	0.80	0.73	0.66
Genitourinary	1.06	1.00	0.93	0.87	0.80	0.74	0.67
Unintentional injuries	1.09	1.01	0.93	0.85	0.77	0.69	0.62
Intentional injuries	1.13	1.03	0.93	0.84	0.74	0.65	0.56
Maternal	0.97	0.95	0.93	0.91	0.88	0.85	0.82

For peer review only

For peer review only - http://bmjopen.bmj.com/site/about/guidelines.xhtml

(B)

	Ratio of mortality by cause, compared to current mortality rate (at 0%)						
**Percentage point change in FHS coverage:**	**-20%**	**-10%**	**0%**	**10%**	**20%**	**30%**	**40%**
All causes	1.15	1.11	1.07	1.03	0.99	0.95	0.91
Infections (excluding HIV, TB, malaria, NTDs)	1.14	1.11	1.07	1.03	0.99	0.96	0.92
HIV	1.11	1.09	1.07	1.05	1.02	1.00	0.98
TB, malaria, NTDs	1.07	1.07	1.07	1.07	1.08	1.08	1.09
Respiratory	1.14	1.10	1.07	1.03	1.00	0.97	0.94
Nutrition	1.12	1.09	1.07	1.04	1.02	0.99	0.97
Neoplasms	1.14	1.11	1.07	1.03	0.99	0.95	0.91
Nervous system	1.09	1.08	1.07	1.06	1.05	1.04	1.04
Endocrine	1.14	1.11	1.07	1.03	0.99	0.96	0.92
Mental/substance use	1.14	1.10	1.07	1.03	1.00	0.96	0.93
Stroke	1.17	1.12	1.07	1.02	0.96	0.91	0.85
Heart disease	1.14	1.11	1.07	1.03	0.99	0.96	0.92
Other cardiovascular	1.17	1.12	1.07	1.02	0.97	0.92	0.86
Digestive	1.14	1.10	1.07	1.03	1.00	0.96	0.92
Genitourinary	1.13	1.10	1.07	1.04	1.00	0.97	0.94
Unintentional injuries	1.17	1.12	1.07	1.02	0.97	0.92	0.86
Intentional injuries	1.20	1.14	1.07	1.00	0.93	0.86	0.78
Maternal	1.04	1.05	1.07	1.09	1.10	1.13	1.15

For peer review only - http://bmjopen.bmj.com/site/about/guidelines.xhtml
For peer review only

Supporting Information Figure 1: Hazard ratios for FHS users (compared to non-users) by cause of death.

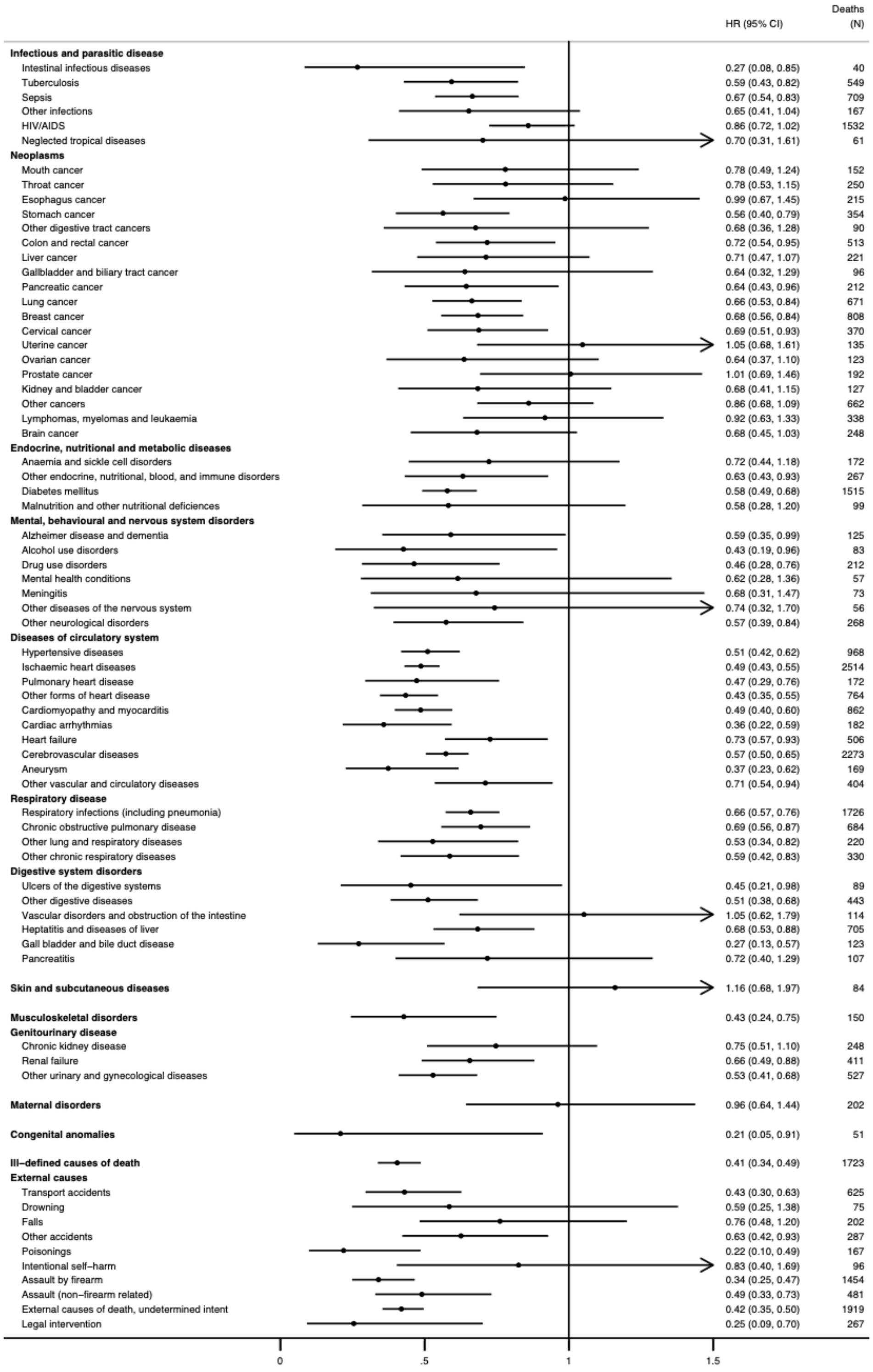

For peer review only - http://bmjopen.bmj.com/site/about/guidelines.xhtml

Supporting Information Figure 2: Uncertainty estimates around mean projected variations in (A) all-cause crude mortality, (B) all-cause age-standardized mortality, (C) infant mortality and (D) under-5 mortality given different levels of Family Health Strategy (FHS) program primary care coverage. See Table 1 for current coverage levels corresponding to a 0% change on the x-axis. Mean values are population-weighted across all 15 Brazilian cities.

(A)

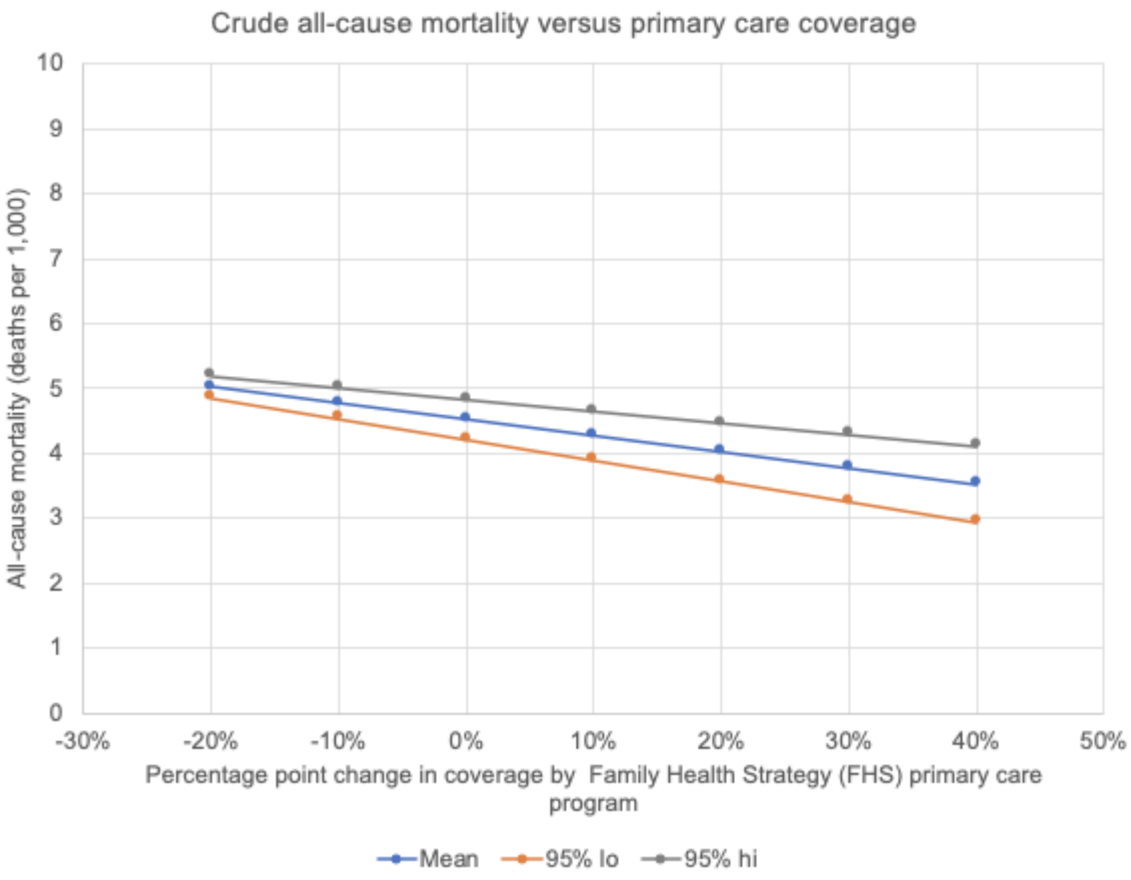

(B)

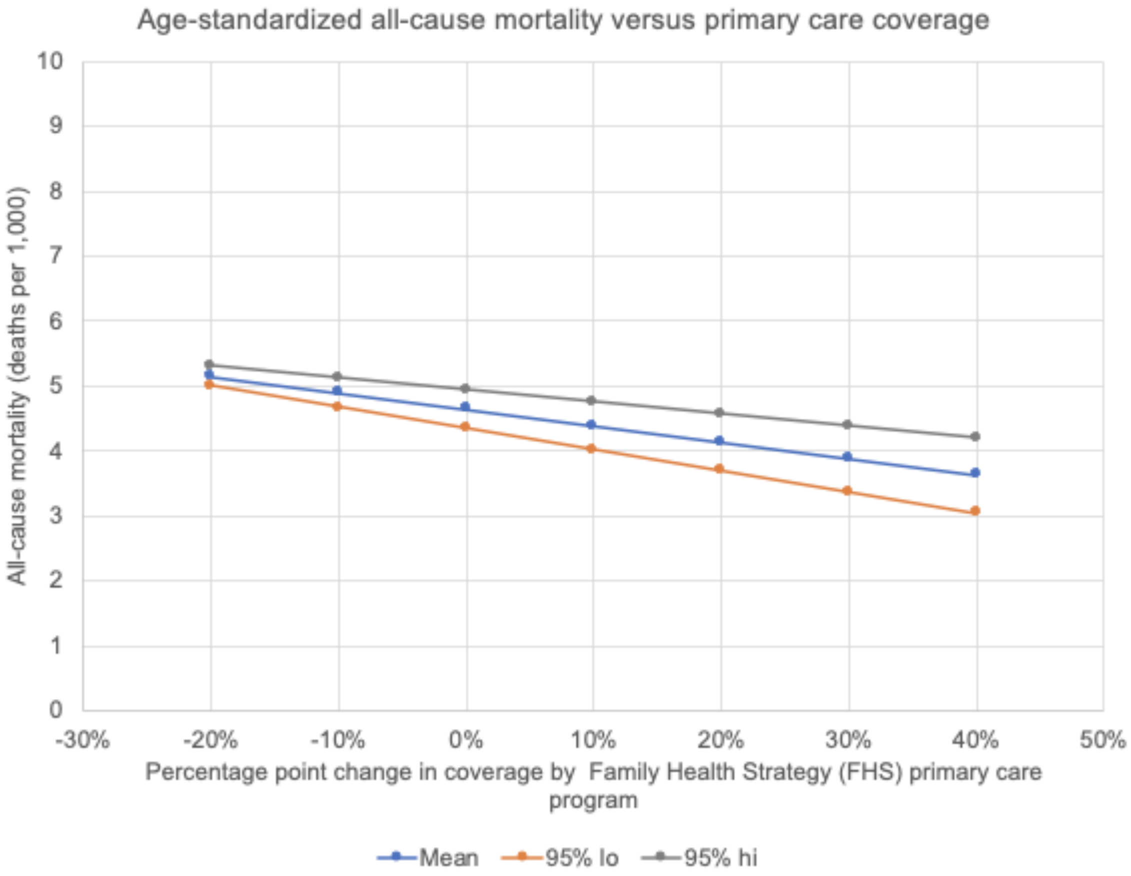

For peer review only - http://bmjopen.bmj.com/site/about/guidelines.xhtml

(C)

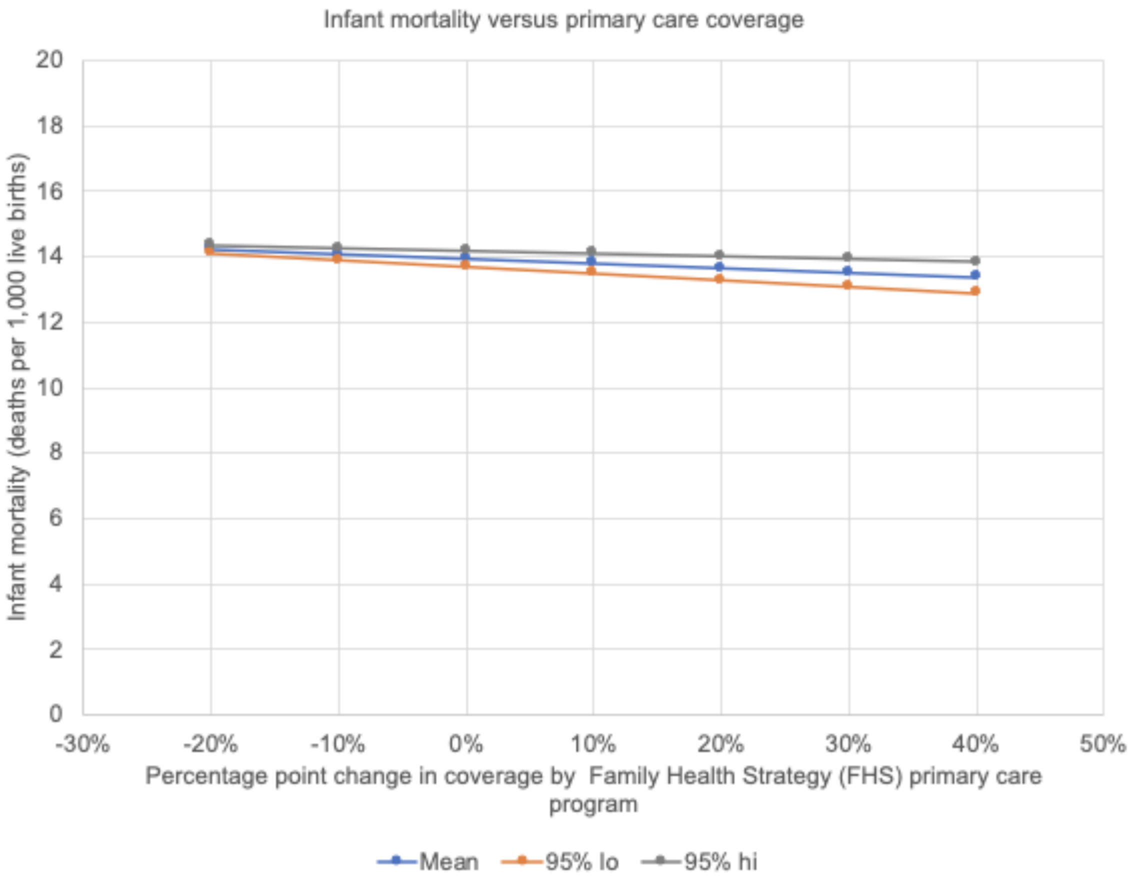

For peer review only - http://bmjopen.bmj.com/site/about/guidelines.xhtml

(D)

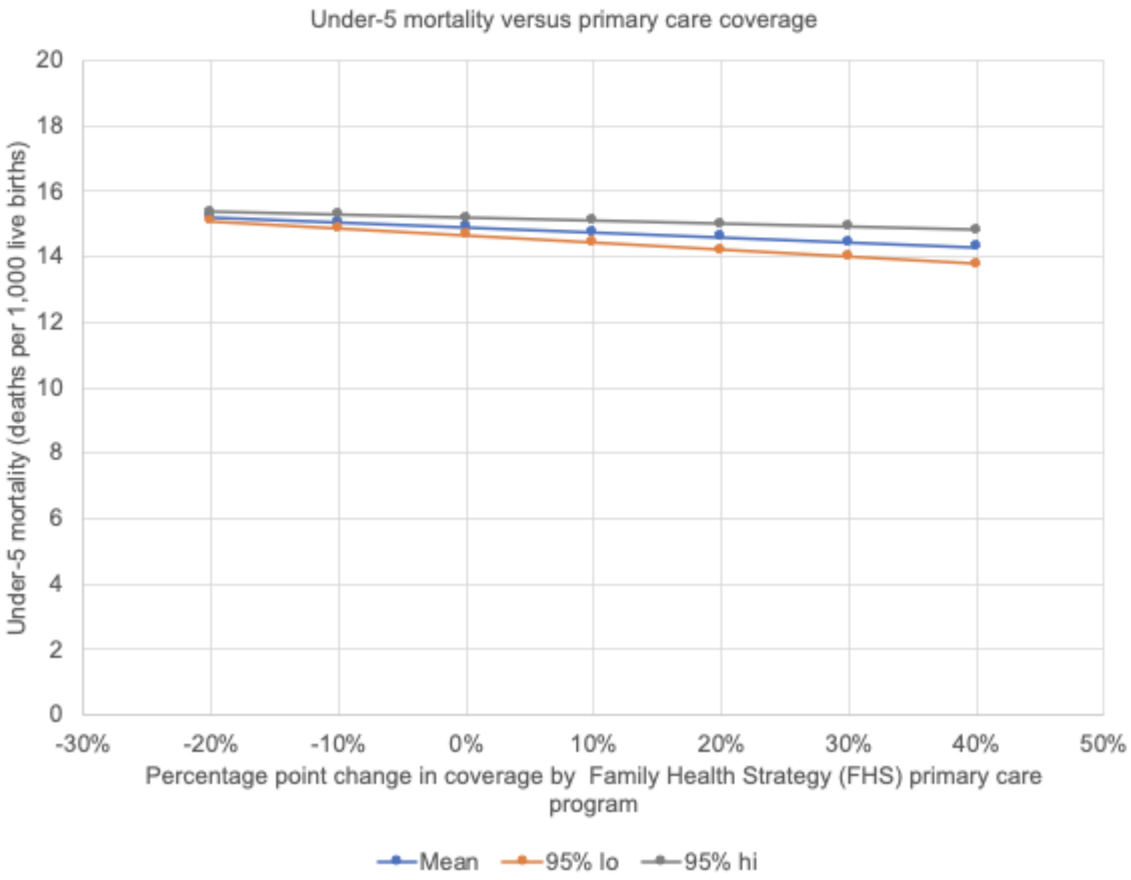

ew only

For peer review only - http://bmjopen.bmj.com/site/about/guidelines.xhtml
